# VPS26 Moonlights as a β-Arrestin-like Adapter for a 7-Transmembrane RGS Protein in *Arabidopsis thaliana*

**DOI:** 10.1021/acs.biochem.4c00361

**Published:** 2024-10-28

**Authors:** Fei Lou, Wenbin Zhou, Meral Tunc-Ozdemir, Jing Yang, Vaithish Velazhahan, Christopher G. Tate, Alan M. Jones

**Affiliations:** † Department of Biology, The University of North Carolina at Chapel Hill, Chapel Hill, North Carolina 27599, United States; ‡ MRC Laboratory of Molecular Biology, Francis Crick Avenue, Cambridge CB2 0QH, U.K.; § Gonville and Caius College, University of Cambridge, Cambridge CB2 1TA, U.K.; ∥ Department of Pharmacology, The University of North Carolina at Chapel Hill, Chapel Hill, North Carolina 27599, United States

## Abstract

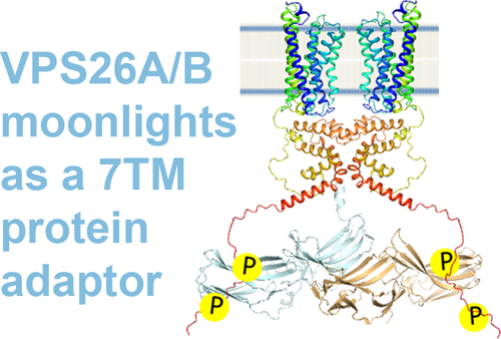

Extracellular signals perceived by 7-transmembrane (7TM)-spanning receptors initiate desensitization that involves the removal of these receptors from the plasma membrane. Agonist binding often evokes phosphorylation in the flexible C-terminal region and/or intracellular loop 3 of many 7TM G-protein-coupled receptors in animal cells, which consequently recruits a cytoplasmic intermediate adaptor, β-arrestin, resulting in clathrin-mediated endocytosis (CME) and downstream signaling such as transcriptional changes. Some 7TM receptors undergo CME without recruiting β-arrestin, but it is not clear how. Arrestins are not encoded in the *Arabidopsis thaliana* genome, yet *Arabidopsis* cells have a well-characterized signal-induced CME of a 7TM protein, designated Regulator of G Signaling 1 (AtRGS1). Here we show that a component of the retromer complex, Vacuolar Protein Sorting-Associated 26 (VPS26), binds the phosphorylated C-terminal region of AtRGS1 as a VPS26A/B heterodimer to form a complex that is required for downstream signaling. We propose that VPS26 moonlights as an arrestin-like adaptor in the CME of AtRGS1.

## 1 Introduction

Extracellular ligands bind membrane-bound 7TM receptors leading to a conformational change of the receptor that is propagated to cytoplasmic signal transducers and amplifiers.^1,2^ Decoupling from one transducer by decreasing the receptor pool size on the membrane changes response behavior to become hyposensitive to the cognate signal but also may couple to a different transducer to propagate signaling with separate implications.^3^ Ligand binding may also initiate phosphorylation of serine clusters on the cytoplasmic C-terminus and/or intracellular loop 3 (ICL3) of G-protein-coupled receptors (GPCRs) by receptor kinases (GRKs), which consequently recruit β-arrestins that facilitate clathrin-assembly and subsequent clathrin-mediated endocytosis (CME).^4^ Some GPCRs internalize via sterol-dependent endocytosis (SDE),^56^ and others, such as AtRGS1, the subject of this work, use both CME and SDE.^55^

**Figure 1:**
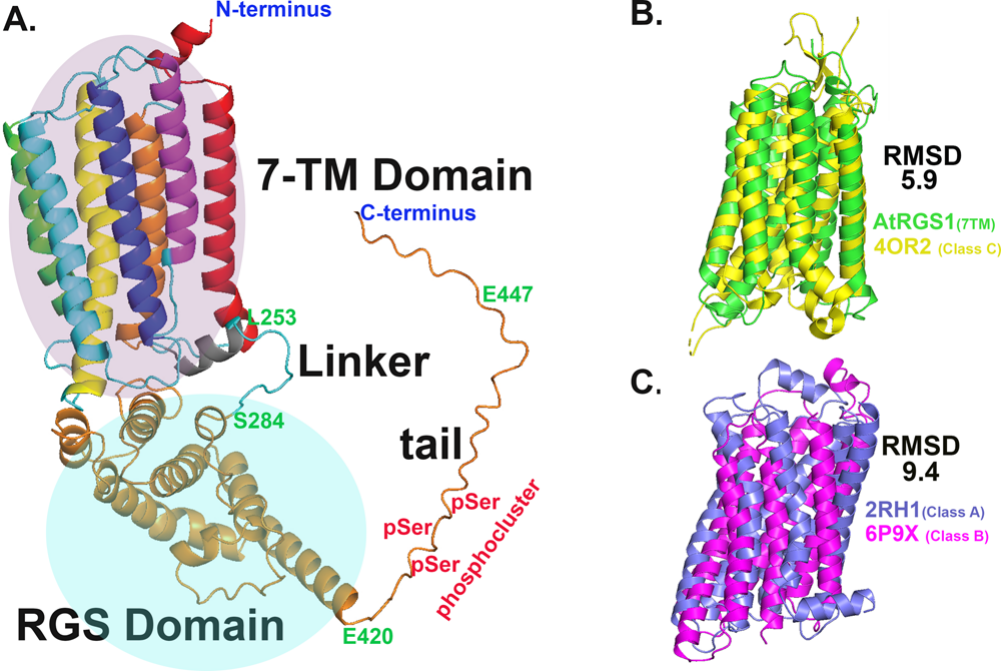
Alphafold2 model of AtRGS1 and its 7-TM domain is structurally most similar to a class C GPCR. (A) Model of AtRGS1 consists of four domains labeled in black: (1) 7-transmembrane (7-TM) domain (highlighted in magenta), (2) RGS domain (highlighted in cyan) is connected by (3) a linker, and (4) a tail that may be disordered. Phosphorylated serines are approximated as indicated with a red “pSer”. Important residues indicating the ends of the proteins used in this study are indicated with positions in green and blue letters. The AtRGS1 constructs used in this study and indicated in Figure 2A are the C-tail, delimited by residues A421 to G459; the truncated tail (delta C- or pC-tail), delimited by A421 to E447; and the RGS -box + C-tail (designated C-domain no tail in Figure 2A), delimited by S284 to G459. The carboxyl-terminal domain lacking the tail (C-domain no tail in Figure 2A) is delimited by S284 to E420. AtRGS1 used for the Mass Photometry experiments in Figure 3 lacked the C-tail and is described in greater detail below. (B) AlphaFold2 model for the AtRGS1 7TM domain (green) is overlaid with the X-ray structure of the human class C GPCR metabotrophic glutamate receptor 1 (PDB 4OR2, yellow). (C) The cryo-electron microscopy structure of class B human corticotropin-releasing hormone receptor 1 (PDB 6P9X, magenta) is overlaid with the crystal structure of the class A human β2 adrenergic GPCR (PDB 2HR1, blue). Relative Mean Square Distances (RMSD) between the entire lengths of these two structures is indicated.

Mammalian arrestins are multifunctional endocytic adaptors^5^ and signal transducers^6,7^ that are comprised of four unique proteins in two subclasses^8^: visual β-arrestins (Arr1and Arr4) that are adaptors for rhodopsin, and nonvisual β-arrestins (Arr2 and Arr3, also known as β-arr1 and 2, respectively) that are adaptors to other GPCRs.^9^ GRK-mediated phosphorylation of GPCRs, typically in unstructured regions of ICL3 and/or the C-terminus, allows binding.^10−13^ β-arrestin coupled to a GPCR can associate with Adaptor Protein complex 2 (AP2) and clathrin for CME. Additionally, β-arrestins may have multiple conformational states to facilitate binding on different phosphorylation clusters or patterns on the C-terminus of receptors.^14^ β-arrestins interact with GPCRs at several sites, including the cytoplasmic face of the 7-TM core,^11,15^ cytoplasmic helix 8 (see gray helix in Figure 1A),^16^ and the phosphorylated tail.^6^ Mammalian GPCRs bound to agonists couple to heterotrimeric G proteins bound to GDP promote a conformational change that facilitates the exchange of GDP for GTP, thus activating the G protein. In contrast, GPCRs are not utilized by plant cells, because their single canonical Gα subunit exchanges GDP for GTP without requiring any other factor like a GPCR.^17−19^ Instead, the plant G protein complex is kept in its inactive state, in part, by a seven-transmembrane (7TM) Regulator of G Signaling (RGS) protein. The prototype 7TM RGS protein is AtRGS1 from *Arabidopsis thaliana*.^20^ In lieu of a known structure, we produced a model for AtRGS1 as described in Material and Methods using Alphafold2.^56^ The model of AtRGS1 predicts with high confidence (Figure S1A) a GPCR-like, 7TM-helical bundle and an intracellular, conserved RGS domain (Figure 1A). The model predicts with less confidence the structure of a linker region between the 7TM bundle and the adjacent RGS catalytic domain (RGS domain) and the structure of the C-terminal tail, possibly because the linker and tail are intrinsically disordered. There is credible sequence homology in ICL2 with the cyclic AMP GPCR cAR1 in slime mold, but cAR1 has functions that are independent of G proteins because cAR1 orthologs are found in ancient organisms that lack G proteins^21^ yet contain arrestin-like proteins.^8^ Cargo recognition utilizes different motifs and these are common in endocytic receptors.^22^ For example, AtRGS1 contains a “tyrosine motif”^23^ located in AtRGS1 at residue Y112 in the second intracellular loop^20^ exactly where it is also located in numerous GPCRs.

Structure prediction by Alphafold2 is not perfect,^57^ and there will undoubtedly be differences between the model and an experimentally determined structure when one becomes available. However, the typical difference between an Alphafold model in regions of high certainty (pLLDT > 80) and an experimentally determined structure is about 1–2 Å RMSD,^58^ which means that the overall fold is predicted well, although the position of individual amino acid side chains should probably not be considered as accurate. A critical evaluation of the structural predictions corroborates the accuracy of Alphafold models and recent data show that they are sufficiently accurate for in silico drug screening.^59^ The utility of Alphafold2 models thus gives us the opportunity to compare the overall predicted architecture of AtRGS1 with those of other receptors to see if there are any indications of similarity.

Like GPCRs, AtRGS1 internalizes upon treatment with signals, notably with a sugar metabolite^24^ and a 22-amino acid peptide (flg22) released from bacteria flagella that is perceived by the plant host to recognize the presence of a potential pathogen.^25^ AtRGS1 internalization induced by flg22 is dependent on phosphorylation of specific serine residues on the cytoplasmic C-terminus by receptor-like kinases^26^ and With-No-Lysine kinases.^27^ We showed that this internalization of AtRGS1 is genuine endocytosis as opposed to simple anterograde trafficking by the retromer because it depends on^25^ : (1) clathrin heavy chain, (2) the clathrin adaptor, AP2m, and (3) a functional cargo-binding motif. (4) Moreover, AtRGS1 internalization is sensitive to the endocytosis inhibitors tyrphostin A23 and ES9–17.^28^ We also showed that flg22-induced endocytosis of AtRGS1 requires: (5) a functional G protein complex including the presence of the atypical extra-large G protein XLG2,^29,30^ (6) an intact carboxy-terminal tail, (7) a series of phosphorylated serines in a cluster in the tail near the RGS domain, (8) a functional BAK1 to phosphorylate these serines in the phosphocluster, (9) FLS2, and, (10) differently than its role in the retromer as will be described below, a Vacuolar Sorting Protein 26 (VPS26) heterodimer.

The human VPS26 protein specifically contains an arrestin-like fold and has a high structural similarity with β-arrestins despite lacking strong sequence similarity. However, VPS26 is already well characterized as a subunit of the retromer complex where it recognizes cargo, resulting in anterograde trafficking from the endosomal compartments to the plasma membrane or Golgi in humans^31^ and in *Arabidopsis*.^32^ Three *Arabidopsis* VPS26 proteins (AtVPS26a, gene At5g53530; AtVPS26b, gene At4g27690; AtVPS26-LIKE, gene At1g48550) have sequence similarity to vertebrate VPS26 and structural similarity to β-arrestin proteins, thus providing a logical starting point for elucidating the AtRGS1 internalization mechanism. The CME of AtRGS1 requires strictly both VPS26A and VPS26B implying that a VPS26A/B heterodimer is involved in AtRGS1 endocytosis^25^ whereas, in contrast, VPS26 forms a homodimer as part of the retromer.^33^ Despite the fold of VPS26 being similar to that of β-arrestins, there are a number of critical differences in the functionality of the proteins. VPS26 does not form an α-helix in the N-domain, it also lacks the polar core between the two lobes of the structure and, finally, it lacks the “three-element interaction” between the first β-strand, the α-helix, and the C-terminus.^60-63^ The latter two elements are critical in maintaining the inactive basal conformation of β-arrestins, and therefore, differences in the mode of interaction and dynamics of VPS26 compared to β-arrestins are expected.

There is a cluster of phosphorylated residues in the C-terminal tail of AtRGS1 that is essential for flg22-induced CME.^34^ Upon activation by flg22, FLS2 receptor kinase rapidly recruits its coreceptor BAK1 receptor kinase to form an active complex and initiates immune signaling.^35^ AtRGS1 is phosphorylated by BAK1 and by BIK1,^34^ and this phosphorylation triggers its dissociation from both FLS2 and from the G protein complex within 10 min.^35^ Such derepression enables autoactivation of Gα protein which then dissociates from the Gβγ dimer and the FLS2 receptor complex to promote immune signaling.^34^ We hypothesize that during this time, the VPS26A/B heterodimer acts as an arrestin-like adaptor and interacts with the AtRGS1 phosphorylated C tail where it dimerizes or oligomerizes AtRGS1 to initiate endocytosis. Activation of FLS2 by flg22 induces a BIK1/PBL-mediated phosphorylation of the AtRGS1 tail at and around Ser428^36^ and Ser431^34^ to promote AtRGS1 dissociation from the FLS2/G-protein complex.

## 2 Materials and Methods

### 2.1 Modeling and Binding Predictions

The model of AtRGS1 (UniProt: Q8H1F2) is derived from the Alphafold protein structure database. (https://alphafold.ebi.ac.uk/entry/Q8H1F2). The VPS26A (UniProt: Q9FJD0) and VPS26B (UniProt: Q9T091) models are derived from the Alphafold protein structure database (https://alphafold.ebi.ac.uk/entry/Q9FJD0; https://alphafold.ebi.ac.uk/entry/Q9T091). The dimer models are predicted through Alphafold2 by inputting the sequence for the proteins (https://github.com/sokrypton/ColabFold/). PRODIGY https://wenmr.science.uu.nl/prodigy/ was used to predict the *K*_d_ between proteins by entering the models obtained from Alphafold2. Temperature is set at 25 °C.

### 2.2 Peptides Used in This Study

The AtRGS1 peptides used in this study were as follows (purity indicated):

- C tail: GGG-A_421_MHKEGYSFSSPRLSSVQGSDDPFYQEHMSKSSRCSSPG_459_ (99%)
- pSer C tail: GGG-A_421_MHKEGY**pS**_**428**_FS**pS**_**431**_PRL**pS**_**435**_SVQGSDDPFYQEHMSKSSRCSSPG_459_ (87%)
- Δ*p*Ser C tail: GGG-_421_MHKEGY**pS**_**428**_FS**pS**_**431**_PRL**pS**_**435**_SVQGSDDPFYQE_447_
- Δ*C* tail: GGG-A_421_MHKEGY**S**FS**S**PRL**S**SVQGSDDPFYQE_447_

The C-tail peptides were synthesized at the UNC Peptide Core Facility. The truncated (Δ) peptides were prepared at the Tufts University Peptide Core Facility.

### 2.3 Protein Expression and Purification

For protein expression, plasmids described below containing 8 × His sumo-VPS26A and 8 × His sumo-VPS26B were transformed into BL21 (DE3) cells. LB medium was inoculated with Amp and grown overnight with constant shaking at 37 °C. In large-scale culture, overnight culture (2% vol) was inoculated with 1 L of the LB medium in 2.5 L flasks. Incubation was done at 37 °C and 225 rpm. At OD600 = 0.6–0.8, the incubator temperature was lowered to 18 °C and it was grown for 30 min. Protein expression was induced using 0.5 mM IPTG at 18 °C for 16 h.

All purifications were performed at 4 °C. Cell pellets were resuspended in extraction buffer (25 mM Tris-HCl pH 8.0, 150 mM NaCl, 0.25 mg/mL Lysozyme, 1 × protease inhibitor cocktail, 5 mM TCEP) and mixed for 30 min at 4 °C. The suspension was sonicated (Sonic Dismembrator, Model 550, Fisher Scientific, power level 5, 10 s on, and 20 s off for 30 min) to disrupt the cells. The lysate was centrifuged at 30,000 × *g* for 40 min, and then the soluble fraction was collected and the supernatant filtered through a 0.45 μm filter. Then, it was incubated with nickel resin for 30 min at 4 °C. The resin was washed with washing buffer (extraction buffer above except with neither lysozyme nor Thesit plus 50 mM imidazole) and eluted with elution buffer (washing buffer supplemented with 250 mM imidazole (Sigma-Aldrich)). The His-sumo tag was cleaved by ULP1 protease, followed by a second nickel chromatography. The eluted proteins were run on a size exclusion column (Superdex 200 10/300 GL, GE Healthcare) with a running buffer (1 × PBS). Aliquoted protein samples were snap-frozen by liquid nitrogen and stored at −80 °C.

His- and GST-tagged RGS + Ct (AtRGS1, residues from 284 to 459) were cloned into the pDEST15 destination vector as previously described (36, 63). AtRGS1 C tail deletion was cloned into the pET 21b(+) vector. Plasmids were transformed into ArcticExpress RP cells (Agilent Technologies). In large-scale culture, 0.5 L of LB medium in 2.5 L flasks were incubated at 37 °C, 225 rpm. At OD600 = 0.6–0.8, protein expression was induced using 0.5 mM IPTG at 12 °C for 16 h. All purifications were performed at 4 °C. Cell pellets were resuspended in extraction buffer (25 mM Tris-HCl pH 8.0, 150 mM NaCl, 2 mM MgCl_2_, 20 μM GDP, 5 mM 2-mercaptoethanol, 1 mM PMSF, 0.25 mg/mL Lysozyme, 0.1% Thesit (Sigma, 88315), 1× protease inhibitor cocktail, 5 mM TCEP) and mixed for 30 min at 4 °C. The suspension was sonicated for 30 min (10 s on and 20 s off) to disrupt the cells. The lysate was centrifuged at 30,000 × *g* for 40 min, and then the soluble fraction was collected and incubated with nickel resin for 30 min at 4 °C. The resin was washed with washing buffer (extraction buffer above except with neither lysozyme nor Thesit plus 50 mM imidazole) and eluted with elution buffer (washing buffer supplemented with 250 mM imidazole). The eluted proteins were subjected to size exclusion chromatography (Superdex 200 10/300 GL, GE Healthcare) with Running Buffer (20 mM Tris-HCl, pH 7.5, 50 mM NaCl, 10 mM MgCl2, 5 mM TCEP). Aliquoted protein samples were snap-frozen by liquid nitrogen and stored at −80 °C. The His-GST tag was cleaved by TEV protease.

### 2.4 Mass Photometry

All data were acquired using a OneMP mass photometer (Refeyn Ltd., Oxford, UK). Microscope coverslips (No. 1.5, 24 × 50 and 24 × 24 mm2, VMR) were cleaned by sequential sonication in 50% isopropanol (HPLC grade)/Milli-Q H2O, and Milli-Q H2O (5 min each), followed by drying with a clean nitrogen stream. Fresh aluminum foil was folded around an A4-sized cutting board. Individual 24 × 24 coverslips were taped using two strips of double-sided tape and cut from the foil using a scalpel blade. Each excised 24 × 24 coverslip was joined, tape side down, in the center of a 24 × 50 coverslip, and stored prior to use as illustrated by.^37^

Immediately prior to mass photometry measurements, protein stocks were diluted directly in stock buffer (unless stated otherwise). Typical working concentrations of protein complexes were 5–25 nM depending on the dissociation characteristics of individual assemblies. Each protein was measured in unused flow chambers. Fresh buffer first flowed into the chamber until the focal position was identified and secured in place with an autofocus system based on total internal reflection for the entire measurement. For each acquisition, 15 μL of diluted protein was introduced into the flow chamber and, following autofocus stabilization, recorded for either 60 or 90 s duration. Each sample was measured at least three times independently.

### 2.5 Quantitative Binding Analyses by Microscale Thermophoresis (MST)

Measurements for equilibrium binding were performed using 20 nM fluorescence-labeled protein using *Monolith NTTM Protein Labeling Kit RED-NHS* and *RED-MALEIMIDE* (Nanotemper Technologies). The dye/protein ratio used was 10:1. Experiments were conducted at 25 °C and in “MST Buffer” [PBS pH 7.4, 0.05% Tween-20]. The protein sample was centrifuged for 30 min (21,000 × *g*, 4 °C) before loading the capillaries. For thermophoresis measurements, ligand and labeled protein samples were mixed at a ratio of 1:1 for each of the ligand dilution series. After 10 min of incubation at room temperature, each dilution was loaded into Monolith NTTM MST Premium-coated capillaries (Nanotemper Technologies). A capillary scan was performed with a 40% LED power. Binding curves were fitted to two sets of replicates. For binding experiments, *K*_d_ values were calculated via the *MO. Affinity Analysis V2.3 software*. This software performs a quality control based on the levels of starting fluorescence, and the distribution of the signal within the capillaries, an indicator of nonspecific binding. A signal-to-noise ratio above 5 was used as the lower cutoff for a high-confidence call of the data.

### 2.6 Plant Growth

Stably transformed *Arabidopsis* lines expressing GFP-tagged AtRGS1 behind the cauliflower mosaic viral (CaMV) 35S constitutive promoter (*35S:AtRGS1-GFP*), GFP (*35S:GFP)*, YFP-tagged AtRGS1 (*35S:AtRGS1-YFP*), or YFP-tagged AtRGS1 with the phosphoserine cluster mutated to alanine (*35S:AtRGS1(3SA)-YFP*) were sterilized and then stratified in 1-ml liquid  MS medium without sugar at 5 °C for 2 days. After they were treated for 2 h with light, the seeds were covered with aluminum foil and kept in darkness at room temperature for 5 days.

### 2.7 Imaging: Analysis of the Raster Scans Acquired for Numbers and Brightness (N&B) Microscopy

N&B uses the average fluorescence intensity, and the variance in fluorescence, to determine the brightness of particles and their number in an image, thus establishing the oligomeric state of proteins.^38,39^ Elongated hypocotyls were imaged using a confocal laser-scanning microscope (Zeiss LSM 880, http://www.zeiss.com/) with a C-Apochromat 40 × /1.2 NA water immersion objective where GFP is excited at 488 nm. Hypocotyls were mounted on slides in water which was replaced with a solution of either water, 6% d-glucose, or 1 um of flg22 and imaged at 0, 30, and 20 min, respectively. The 256 × 256-pixel regions of interest in hypocotyls were raster scanned with a pixel dwell time of 4.12 μs for 100 frames with a pixel size of 100 nm according to RICS image acquisition techniques. The SimFCS software (https://www.lfd.uci.edu/globals/) was used for the analysis of the raster scans acquired for N&B. The background region of the image was normalized and monomer brightness was determined with the 35S:GFP or 35S:YFP lines. A cursor was used to cover all the pixels in the brightness versus intensity graph. The y-position of the cursor is used to calculate the brightness of the fluorescent protein. The cursor was positioned at the brightness value of monomeric protein. Any pixels within this cursor are monomers. Another cursor of the same size is placed on top of the monomer cursor. Pixels that fall inside this box are homodimers. The percentage of monomer was calculated by dividing the number of monomer pixels by the sum of the pixels of all oligomeric forms (monomer plus homodimer). This was repeated to determine the percentage of homodimer forms.

### 2.8 Real-Time Quantitative Polymerase Chain Reaction (RT-qPCR)

RT-qPCR was performed as described by (36). Briefly, approximately 10 seeds of *Arabidopsis thaliana* were germinated and grown sterilely in 1/2 × MS liquid media supplemented with 1% glucose. The growth conditions were maintained at 23 °C with low light intensity (50 μmol s–1 m–2). After 7 days in culture, the seedlings were subjected to a 2-day starvation period in the dark using 1/2 × MS media. Subsequently, the seedlings were stimulated with 6% d-glucose for 3 h under dark conditions to induce gene expression changes. Total RNA was extracted from the harvested seedlings using the Thermo Scientific GeneJET Plant RNA Purification Mini Kit following the manufacturer’s instructions. The RNA was treated with DNase to remove genomic DNA contamination. Complementary DNA (cDNA) was synthesized from the purified RNA using M-MLV Reverse Transcriptase (Invitrogen) according to the manufacturer’s protocol. Real-time PCR was performed using a HotStar 2XSYBR Green qPCR Master Mix (APExBIO) kit. Each treatment and genotype were analyzed in duplicate as technical replicates. Three biological replicates were conducted for each treatment/genotype, except for the *vps26* single mutants. Each treatment/genotype had two technical replicates.

Gene-specific primers for TBL26 and TUB4 were designed for qPCR analysis:

TBL26 forward, 5′-CGCCATCGAACCTTCGTCAAATTC-3′;
TBL26 reverse, 5′ -TCGTCCATTCAATAGGCAGTTCTGA-3′;
TUB4 forward, 5′-AGAGGTTGACGAGCAGATGA-3′;

## 3 Results and Discussion

### 3.1 Conservation of Structure in AtRGS1 with Animal GPCRs Suggests Shared Function, Including Signal-Induced Endocytosis

Plants have 7TM proteins but there is no requirement that these proteins have the GPCR guanine-nucleotide exchange function to activate G proteins as found in mammals.^36^ However, plant 7TM proteins, including AtRGS1, may share another GPCR function such as coupling to an arrestin-like adaptor to transduce signaling. Thus, before we directly tested the coupling of AtRGS1 to an arrestin adaptor, we asked if AtRGS1 shares an evolutionary history with an animal GPCR because structural homology portends functional homology. Establishing evolutionary history over the great temporal distance of 1.6+ B years that all 7TM proteins share has been difficult with essentially the only progress made was in taxonomy within the opistokonts (animals and fungi clade).^40^ Mammalian GPCRs are well-curated into six classes based on primary sequence alignments, motifs, and ligands.^41^ Unfortunately, sequence divergence even over a short distance is large even among human GPCRs making inferences about homology between human GPCRS and plant and protist 7TM RGS proteins difficult at this time^42^ despite more recent views of electronic distribution similarities found between extant GPCRs and microbial 7TM proteins.^62^ Therefore, to address the question of potential homology between AtRGS1 and a human GPCR, we avoided sequence comparisons and, instead, directly compared 3-dimensional structures and quantitated differences. The AlphaFold2 model for the 7TM domain of AtRGS1 was compared to the known structures of at least one member in each of the human GPCR classes A through D. AtRGS1 is most similar to β_2_AR (a Class A GPCR; PDB 2RH1(43)) and mGlutR (a Class C GPCR; PDB 4OR2), with the latter having an RMSD of 6.0 Å calculated over the entire protein including the unstructured loops (Figure 1B). In comparison, as shown in Figure 1C, a human class C GPCR shows less similarity to a human class A GPCR (mGlutR compared with β_2_AR, RMSD = 9.4 Å). Comparisons between human class A, B, and C GPCRs, and the yeast class D GPCR (Ste2, PDB 7AD3,^44^), indicated that AtRGS1 is more similar to class C and A GPCRs than these classes are to classes B and D (Figure S1B–F). This suggests that the architecture of the AtRGS1 7TM domain shares overall homology to human GPCRs. Although this is suggestive, without considerably greater depth of coverage of structures and more detailed analyses of the comparisons, we do not exclude the possibility that there are only a limited number of 7TM structures that are possible, and all of these are found among the human GPCRs. As such, any 7TM structure from an organism distal to a human phylogenetically could falsely imply an evolutionary relationship with a human GPCR.

### 3.2 C-terminal Region of AtRGS1 Is Crucial for the Binding between AtRGS1 and VPS26A/B

Using indirect quantitation, we showed previously^25^ that AtVSP26A and AtVPS26B (hereafter VPS26A and VPS26B) interact in vivo with AtRGS1 by binding to its cytoplasmic domain (residues L253 to G459, Figure 1). To test this directly, to quantitate the binding affinity, and to map the location of the interacting sites, we used Microscale Thermophoresis (MST) to quantitate the binding of the phosphorylated and unphosphorylated truncated AtRGS1 proteins (Figure 2A). Briefly, MST provides a means to precisely obtain any small change in molecular mass of a fluorescent target that occurs upon binding through measurement of its location and movement in a thermal gradient.^45^ The raw data of the replicated binding curves used to support the reported affinities of Figure 2A are provided in Supporting Information (Figures S2–S20).

**Figure 2:**
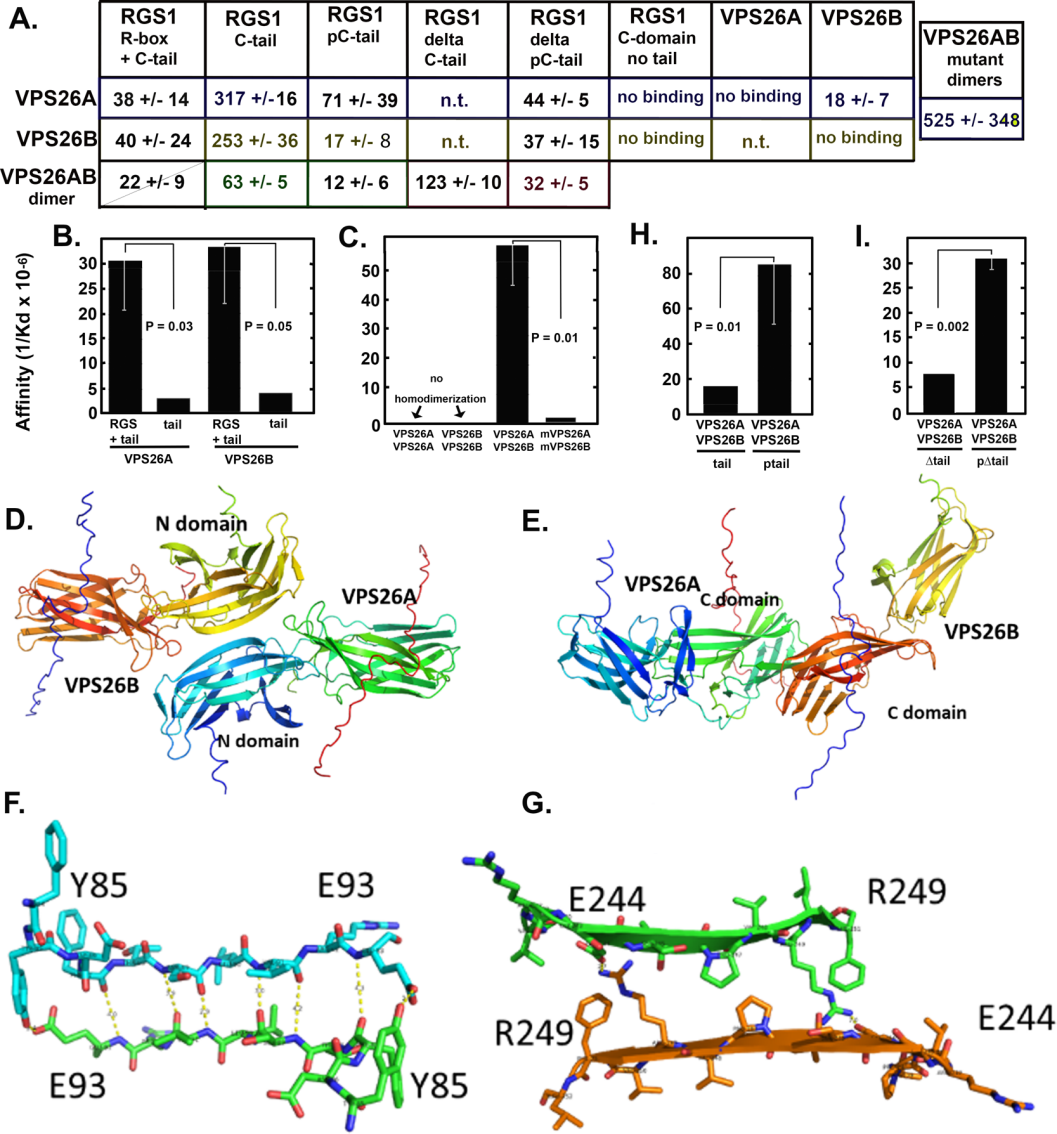
AtVPS26A/B (VPS26A/B) head-to-head, heterodimer binds the tail of AtRGS1 in a phosphoserine-dependent manner. (A) Summary of the binding affinity (average *K*_d_ (nM) from 2 to 4 independent experiments ± SD) among VPS26A, VPS26B, and VPS26 heterodimer to tested versions of AtRGS1 (RGS1) as indicated: RGS1 R-box + C tail, truncated AtRGS1 amino acids S284-G459 contains the RGS domain and the full length unphosphorylated C-terminal tail region; RGS1 C-tail, unphosphorylated C-terminal tail, amino acids A421-G459 chemically synthesized peptide; RGS1 pC-tail, phosphorylated (serines 428, 431, 435) C-terminal tail amino acids A421-G459 synthesized peptide; RGS1 delta C-tail, truncated C-terminal tail amino acids A421-E447 chemically synthesized peptide; RGS1 delta pC-tail, RGS1 C-tail, phosphorylated (serines 428, 431, 435) truncated C-terminal tail amino acids A421-E447 chemically synthesized peptide; RGS1 C domain no tail, truncated AtRGS1 amino acids L253-E420 contains the linker and the RGS domain (box). Also quantitated were pairs of VPS26 A and B homodimers and the VPS26A/VPS26B heterodimer (indicated as VPS26A/B dimer). The values were determined from the binding isotherms provided in Supporting Information (Figures S2–S20) The values are averages of *K*_d_ values from replicates, and the error shown is the Standard Deviation. Each experiment was replicated at least twice; most of the experiments were replicated at least three times. Experiments using the same batch of purified protein are highlighted in their respective colors. n.t. = not tested. “No binding” means that the *K*_d_’s were so high as no meaningful binding above thermal background could be calculated. The experimental data used to generate the *K*_d_s along with quality control of the samples are shown in Figures S2–S20. (B, C, H, I) The values in the table shown in panel A are used to derive affinity (1/*K*_d_) where *K*_d_ is nM units. Standard deviations of the *K*_d_’s are shown in the table in panel A. (B) Binding affinity of the RGS domain of AtRGS1 plus its full C-terminal tail (RGS+tail) or the tail alone (tail) or the phosphorylated tail (ptail) to full-length VPS26A and VPS26B. See above for amino acid ranges and phosphorylated serines for each of the RGS1 proteins. Variance is provided in A. Gray arrow bars = one SEM below the mean. When error bars are not shown, the SEM is below the resolution of the figure. Student T (unpaired) P values are shown with the indicated comparisons indicated with black lines. (C) Binding affinity of heterodimeric VPS26A/B and mutant VPS26A/B heterodimers. The point mutations at the interface shown in panel F are described in the main text. Homodimers were not detectable, so the values are indicated as zero. (D) Top row shows AlphaFold2 models of the VPS26A/VPS26B heterodimer as head-to-head (N-domains) and (E) tail-to-tail (C-domains) heterodimers. (F) Structure in panel D model highlighting the dimer interface. (G) Structure in panel E model highlighting the dimer interface. Indicated are the residues that were mutated for testing interaction affinity as discussed in the text.

Truncated AtRGS1 that contains just the RGS domain and the C-terminal tail (S284 to C terminus) bound VPS26A and VPS26B with similar dissociation constants (34 ± 6 nM and 35 ± 27, respectively) (Figure 2B). The high affinity suggests that heterodimerization is constitutive, given that VPS26 is not found at low levels in the cell. For comparison, AtRGS1 interaction with its Gα subunit partner AtGPA1 has an apparent *K*_d_ of 125 ± 81 nM.^29^ The unphosphorylated tail (residues A421-G459, Figure 1) bound VPS26A and VPS26B with about 10-fold lower affinity (Figure 2B) albeit at nanomolar *K*_d_s (217 and 253 nM, respectively, Figure 2A) suggesting that both the RGS domain and the unphosphorylated tail contribute to the free energy change in this interaction. We speculate that the high observed affinities are either due to the (1) extremely low amount of AtRGS1 in the cell thus assuring interaction or (2) the function requires a stable complex for a reasonably long period, thus the off rate is low. The cytoplasmic domain lacking the tail (L253 to E420, Figure 1) bound neither VPS26A nor VPS26B (Figure 2A) indicating that the tail is critical. The observations of different binding affinities of the phosphorylated/unphosphorylated tail with or without the RGS domain to the VPS26 heterodimer suggest that there are potentially multiple modes of binding in the cell. This is likely to occur when there are multiple different determinants, including both phosphorylated and nonphosphorylated sites. It is also possible that the single VPS26A and VPS26B proteins are not in their native oligomeric states under these conditions. To this point, it should be noted that in these experiments, it is not known if the VPS26 proteins in solution are monomeric or homodimeric or possibly are obligate heterodimers. This is tested in the following section.

### 3.3 VPS26A/B Heterodimer Interface Likely Contains the N Domain β Sheet Stacking between Y85 to E93 Residues

Previously, we used genetic and physical interaction in vivo to show qualitatively that VPS26 functions as a heterodimer.^25^ We thus quantitated the apparent *K*_d_ between VPS26A and VPS26B in a heterodimer in solution and compared it to the interaction between the respective proteins in homodimers (Figure 2C). As expected, the affinity between VPS26A and VPS26B in a heterodimer was strong (*K*_d_ ∼ 18 nM) as measured between fluorescently tagged VPS26A and increasing concentrations of VPS26B. Interactions between either VPS26A alone or VPS26B alone to form the respective homodimers were not detectable (Figure 2A). This indicates that VPS26A and VPS26B are more likely to form a heterodimer than a homodimer which is consistent with the previous in vivo BiFC, split luciferase, and genetic interaction data.^25^

The heterodimer VPS26A/B bound the phosphorylated C-tail of AtRGS1 with ∼5-fold higher affinity than to the unphosphorylated C-tail (Figure 2H). The free energy change in this binding resides largely within 25 residues (A421-E447) because truncation of the 12 terminal residues did not decrease this phosphorylated and unphosphorylated C-tail comparison (Figure 2I).

While there is a structure for the animal VPS26 homodimer in the retromer complex (PDB ID 7BLQ), no physical structure is known for a plant VPS26A/B heterodimer. Therefore, a model was generated using AlphaFold2 to show the potential binding orientation of the heterodimer of VPS26A with VPS26B. There are two potential binding orientations predicted, formed by either the N-domain or C-domain, with both comprising an antiparallel beta-sheet (c.f. Figure 2D,E). PRODIGY^46^ was used to predict the *K*_d_ of binding between the proteins for each model. The predicted *K*_d_ of the N-domain stacking binding pattern was ∼26 nM, similar to the experiment value, ∼18 nM (Figure 2A). In contrast, the predicted *K*_d_ for the C domain stacking binding pattern was ∼1500 nM, a 50-fold decrease in affinity compared to the N-domain stacking model. Thus, both the in silico and experimental data suggest that VPS26A and VPS26B form a heterodimer by head-to-head, antiparallel beta-sheet stacking, with an additional hydrogen bond predicted between the side chains of Tyr85 and Glu93 (cf. models in Figure 2F,G).

Our model of the binding of the N-domain stacking predicts that the free energy change is largely due to at least seven hydrogen bonds between the nine amino acid residues 85–93 (Figure 2F). This equivalent Y85-E93 region is conserved in both plant and animal VPS26 families. The VPS26 dimer crystal structure shows a similar binding surface between the two antiparallel Y85-E93 regions^33^ although the model of the *Arabidopsis* VPS26A/B interface has two additional hydrogen bonds. Based on our model and the animal VPS26 dimer interface, we generated a VPS26B Y85A/E93A mutant to abolish two of these hydrogen bonds between the respective side chains and quantitated the apparent *K*_d_ using MST. The VPS26B^Y85A/E93A^ mutant heterodimerized about 10-fold lower compared to VPS26B wildtype. This is consistent with our model of the VPS26A/B heterodimer (Figure 2D).

**Figure 3:**
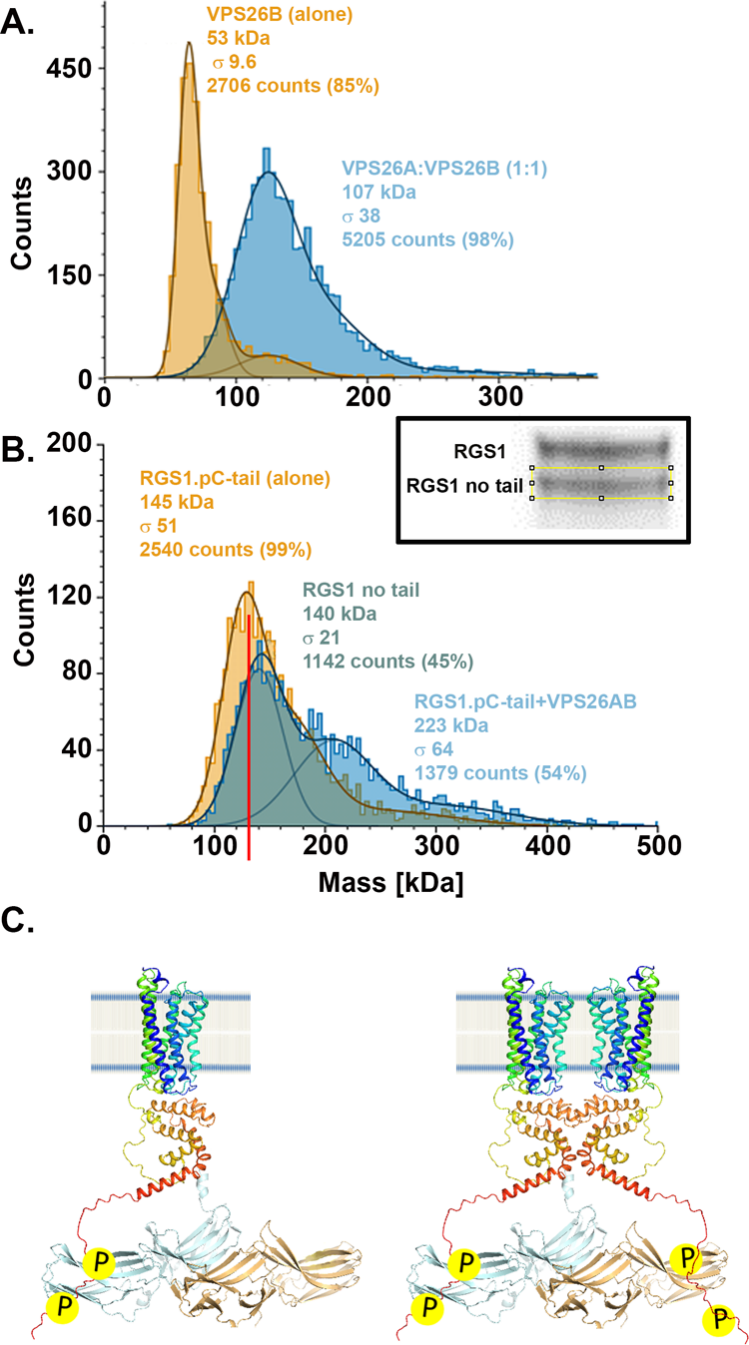
Mass photometry of VPS26A/B and AtRGS1 pCtail complex. (A) Molecular mass distribution histogram of VPS26B and VPS26A/B (ratio 1:1). (B) pC tail and the complex of AtRGS1 (RGS1) with VPS26AB. The size of the AtRGS1 complex increased in the presence of the VPS26AB heterodimer. This is a mixed population of AtRGS1 with only ∼50% of the population containing the C-terminal tail (see Inset). The theoretical molecular weights of the components are VPS26A, 50 kD; VPS26B, 50 kD; RGS1, 80 kD; RGS1-pCtail, 83 kD; pC tail, 3.5 kD. The solid line represents major species fit with Gaussian functions and the width of the fitted peak is given by Gaussian Curve (σ represents the standard deviation). The sum of the number of counts under the fitted peak is expressed as the number of counts and the percentage of counts determined by the counts located in the bound or unbound populations of AtRGS1). (C) As shown in panel A, VPS26A and VPS26B form a heterodimer in vitro with high affinity (Figure 2C). Left, a single AtRGS1 in detergent modeled with one VPS26A/B heterodimer, has a molecular mass consistent with the peak observed in panel B (223 kDa). This model also shows binding of a free phosphorylated tail presumably bound under the conditions used for the MP experiment. Right, dimeric AtRGS1 is modeled bound to a VPS26A/B heterodimer. Because each VPS26A or VPS26B has a binding interface with the AtRGS1 C tail, it is expected that this valence of two increases the overall affinity of the heterodimer compared to that of either monomer.

Mass photometry (MP) was performed to investigate the VPS26A/B heterodimer formation in solution at equilibrium (Figure 3A). MP showed that VPS26A and VSP26B exist predominantly as monomers with a nearly monodisperse peak at 53 kDa. At a 1:1 ratio of VPS26A and VPS26B, MP showed a monodisperse, 107 kDa complex consistent with a heterodimer.

### 3.4 VPS26A/B Heterodimer Forms a Complex with Monomeric and Dimeric AtRGS1 In Vitro

Full-length purified AtRGS1 was prepared from recombinant AtRGS1 with the C-tail truncated and a sortase A ligation site added to the C-terminus.^47^ To this site was ligated a phosphorylated peptide with a sequence identical to the C-tail of AtRGS1. The phosphorylated residues lie within the phosphocluster (see Figure 1A) and are Ser_428_, Ser_431_, and Ser_435_. The efficiency of ligation was ∼50% (Figure 3B, inset); thus, the final sample was a mixture of full-length and truncated AtRGS1. MP revealed that the VPS26A/B heterodimer interacts with full-length AtRGS1 (Figure 3B), but not with the truncated AtRGS1. Purified AtRGS1 in its detergent micelle has an apparent molecular weight of 145 kDa based on its calibrated elution volume. The error in mass associated with this method is ∼5%. When the VPS26A/B heterodimer is added, the monomer population decreased (Figure 3B, green population), and the distribution of a higher-order complex shifted to a higher average weight of 223 kDa, consistent with one AtRGS1 in detergent binding to one VPS26A/B heterodimer (Figure 3C, left). The mass of detergent for a 7TM protein is in the range of 70–110 kDa. Given that the concentration of VPS26 protein was at least as high as the *K*_d_ for the heterodimer binding, we assume that the VPS26A/B heterodimer was the predominant form in these experiments.

Even larger AtRGS1-VPS26 complexes were observed (Figure 3B, blue-colored population). These larger aggregates are consistent with, for example, an AtRGS1 dimer binding to a VPS26A/B heterodimer (Figure 3C, right). The presence of larger aggregates has been previously reported in vivo^24,25,36^ and under the conditions used for the MP experiments we also observe higher-order oligomers of AtRGS1 and VPS26A/B. The question of whether the AtRGS1 dimer is constitutively or inducibly formed is addressed in the next section.

### 3.5 AtRGS1 Dimer Formation Depends on Phosphorylation and the VPS26A/VPS26B Heterodimer

**Figure 4:**
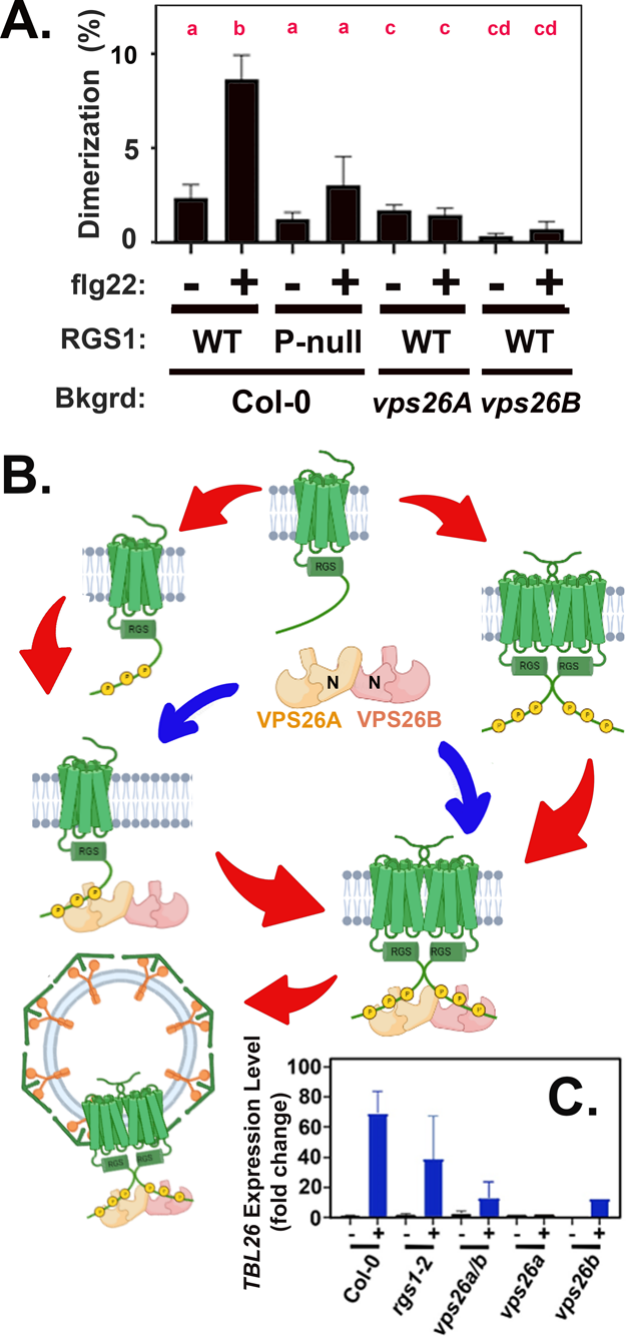
Inducible phosphorylation drives VPS26A/B heterodimer-dependent dimerization of AtRGS1. (A) In the wildtype (Col-0), AtRGS1 exists mainly as a monomer (∼98.5% resting state). flg22 induces dimer formation and increases the dimer percentage from 1.5 to ∼9.2%. Genetic ablation of either the VPS26A (*vps26a*) or VPS26B (*vps26b*) subunit of the VPS26 heterodimer eliminates flg22-induced dimerization. Error bars = Standard Deviation. Letters indicate statistical similar groups (*P* < 0.05) based on ANOVA with Tukey Kramer (B) Scheme for the order of complex formation. Red arrows indicate the flow toward formation of the complex of homodimeric AtRGS1 with heterodimeric VPS26A/B shown at the bottom. Blue arrows indicate two possible places where the VPS26 dimer is recruited to the complex. The right side of the scheme shows AtRGS1 dimer formation preceding VPS26 recruitment, and the left side shows that binding of VPS26 to the AtRGS1 monomer drives homodimer formation. Analogous to β–arrestin binding to animal GPCRs, the complex recruits a clathrin and preendosome formation as shown in the bottom left. Signaling can be initiated from endosomal GPCRs. (C) AtRGS1 mediates expression of the gene Trichome Birefringent Like 26 (*TBL26*) as described previously.^36,50^ Genetic ablation of AtRGS1 reduces *TBL26* expression, while loss of the VPS26A (*vps26aA*), VPS26B (*vps26b*) subunits or loss of the heterodimeric VPS26A/B complex (*vps26a/b*), strongly eliminates *TBL26* expression, error bars = standard deviation.

The mass of the AtRGS1-VPS26A-VPS26B complex determined by mass photometry is consistent with those of some complexes containing an AtRGS1 dimer (Figure 3C). To determine if AtRGS1 dimerization occurs in vivo, we used Number and Brightness (N&B) microscopy,^39^ a fluorescence-based method that measures the apparent average number of molecules and their oligomerization state (brightness) in each pixel from a series of fluorescence microscopy images. In the unphosphorylated resting state, AtRGS1 exists in the plasma membrane of a plant cell mainly as a monomer (∼98.5%; Figure 4A). The addition of the flg22 peptide to plant cells increases the dimer percentage from 1.5 to ∼9% (Figure 4). This is likely a conservatively low value given the dynamics of the reaction (10 s to 3 min^48,49^ and our ability to capture a change with the time resolution of this technique (2–3 min). The raw values of each replicate are provided in Table S1 of Supporting Information).

To ascertain the potential role of AtRGS1 phosphorylation and recruitment of the VPS26A/B heterodimer in AtRGS1 homodimerization, we measured in vivo dimerization of AtRGS1 using N&B microscopy in combination with mutational genetics. Mutation of four serines to alanine in the phosphocluster (Figure 4A, P-null) greatly reduced or eliminated the flg22-induced dimerization of AtRGS1. Genetic ablation of either subunit of the VPS26AB heterodimer also eliminated flg22-induced AtRGS1 dimerization (Figure 4A). Two plausible mechanisms for inducible dimerization are shown in Figure 4B for which the N&B microscopy data are consistent: (a) phosphorylation at the phosphocluster directly drives the formation of the dimer which then recruits the VPS26A/VPS26B heterodimer; (b) phosphorylation does not directly drive AtRGS1 dimerization, but it is directly required for VPS26A-VPS26B recruitment to the phosphorylated C-tail, which results in AtRGS1 dimer formation. We show that either pathway precedes the CME and possibly downstream signaling.

To determine if downstream signaling requires VPS26, gene expression was measured using the inducible, AtRGS1-dependent reporter gene, *TBL26*(50) (Figure 4C). In the absence of either VPS26A or VPS26B, *TBL26* expression was not induced. This suggests that AtRGS1 in the endosome recruited by the VPS26 dimer precedes AtRGS1-mediated downstream signaling at least in part.

VPS26A/B heterodimer acting as a putative arrestin-like adaptor to AtRGS1 contrasts with VPS26 function in the retromer. In the retromer, VPS26 does not bind the cargo directly but indirectly through a cargo adaptor. For example in animals, cargo adaptors to the retromer include sorting nexin-3 (SNX3),^33^ SNX27,^51^ CCC (CCDC22/CCDC93/COMMD),^52^ and endosomal SNX-BAR.^53^ In contrast, we show that the VPS26A/B heterodimer binds AtRGS1 directly (Figures 2A and 3B) in a manner somewhat analogous to β–arrestin binding to some GPCRs, at least to the cytoplasmic tail (Figure 2D).

## 4 Conclusions

Taking the present data together with the body of literature, we speculate that the VPS26A/B heterodimer binds AtRGS1 in a way analogous to the initial interaction between the phosphorylated C-terminus (or ICL3) of a mammalian GPCR and β-arrestin. This suggests that the convex C domain of the VPS26A/B heterodimer, which is rich in positively charged amino acid residues, binds AtRGS1 through the phosphorylated C tail region. The peptide flg22 acts as a signal (no direct proof that flg22 is an AtRGS1 agonist) to activate the phosphorylation pathway through coreceptors BAK1 and FLS2,^54^ resulting in the phosphorylation of the AtRGS1 C-terminal region and recruitment of the VPS26A/B heterodimer. In this process, AtRGS1 dimerization is triggered through the interaction of the VPS26A/B heterodimer with the phosphorylated C-terminal region of AtRGS1 (Ser428 + Ser431 + Ser435). The VPS26 heterodimer will further recruit AP2 and clathrin to the complex and thus induce CME of AtRGS1.^25^ We propose that the VPS26A/B heterodimer is acting outside its established role in the retromer complex and thus moonlights as an adaptor for the endocytosis of AtRGS1, functionally analogous to the role of β–arrestin in facilitating endocytosis and signaling by many GPCRs.

## Supporting Information Available

AlphaFold2 model of AtRGS1 with confidence intervals, Data from each of the experiments utilizing Microscale Thermophoresis (MST) (PDF)

## Accession Codes

The identifiers for the proteins used in this study are as follow: AtRGS1, UniProt: Q8H1F2; AtVPS26A, UniProt: Q9FJD0; AtVPS26B, UniProt: Q9T091.
