# Supplementary material for: VPS26 Moonlights as a β-Arrestin-like Adapter for a 7-Transmembrane RGS Protein in Arabidopsis thaliana
Source: Biochemistry. 2024 Oct 28;63(22):2990–9. doi: 10.1021/acs.biochem.4c00361 (PMC11580166; doi:10.1021/acs.biochem.4c00361)
Supplement: Supplementary file 1 — bi4c00361_si_001.pdf [file bi4c00361_si_001.pdf]

SUPPLEMENTAL INFORMATION

VPS26 moonlights as a β-arrestin-like adapter for a 7-transmembrane RGS protein in *Arabidopsis thaliana*

Fei Lou[1], Wenbin Zhou[1], Meral Tunc-Ozdemir[1], Jing Yang[1], Vaithish Velazhahan[2,3], Christopher G. Tate[2], and Alan M. Jones[1,4,*]

[1]Department of Biology, The University of North Carolina at Chapel Hill, Chapel Hill, NC 27599 USA
[2]MRC Laboratory of Molecular Biology, Francis Crick Avenue, CambridgeCB2 0QH, UK
[3]Gonville and Caius College, University of Cambridge, Cambridge, UK
[4]Department of Pharmacology, The University of North Carolina at Chapel Hill, Chapel Hill, NC 27599 USA

Figure S1 AlphaFold2 model of AtRGS1 with confidence intervals. Supports Figure 1B and C
(A) AlphaFold2 model of AtRGS1 was generated as described in Materials and Methods. AlphaFold II produces a per-residue confidence score using the predicted local distance difference test (pLDDT) between 0 and 100 [34] with regions below 50 predicted may be unstructured in isolation and colored as orange and yellow in the structure. The other colors of the structure indicate the model confidence as indicated. : Dark blue- high (pLDDT > 90) , light blue- Confident (90 > pLDDT > 70), orange - Low (70 > pLDDT > 50), yellow or orange - low (pLDDT < 50). **(B-F)** Overlay of 7-TM domains from the indicated structures: **(B)** human Class GPCR CRT receptor (PDB 6P9X, grey) with human Class A β2 Adrenergic Receptor (PDB 2HR1, blue). **(C)** human Class C GPCR metabotropic receptor (PDB 4OR2, yellow) with human Class A β2 Adrenergic Receptor (PDB 2HR1, blue). **(D)** human Class B GPCR CRT receptor (PDB 6P9X, grey) with C GPCR metabotropic receptor (PDB 4OR2, yellow). **(E)** human Class B GPCR CRT receptor (PDB 6P9X, grey) with yeast Class D STE2 receptor (PDB 7AD3, magenta). **(F)** human Class C GPCR metabotropic receptor (PDB 4OR2, yellow) with yeast Class D STE2 receptor (PDB 7AD3, magenta).

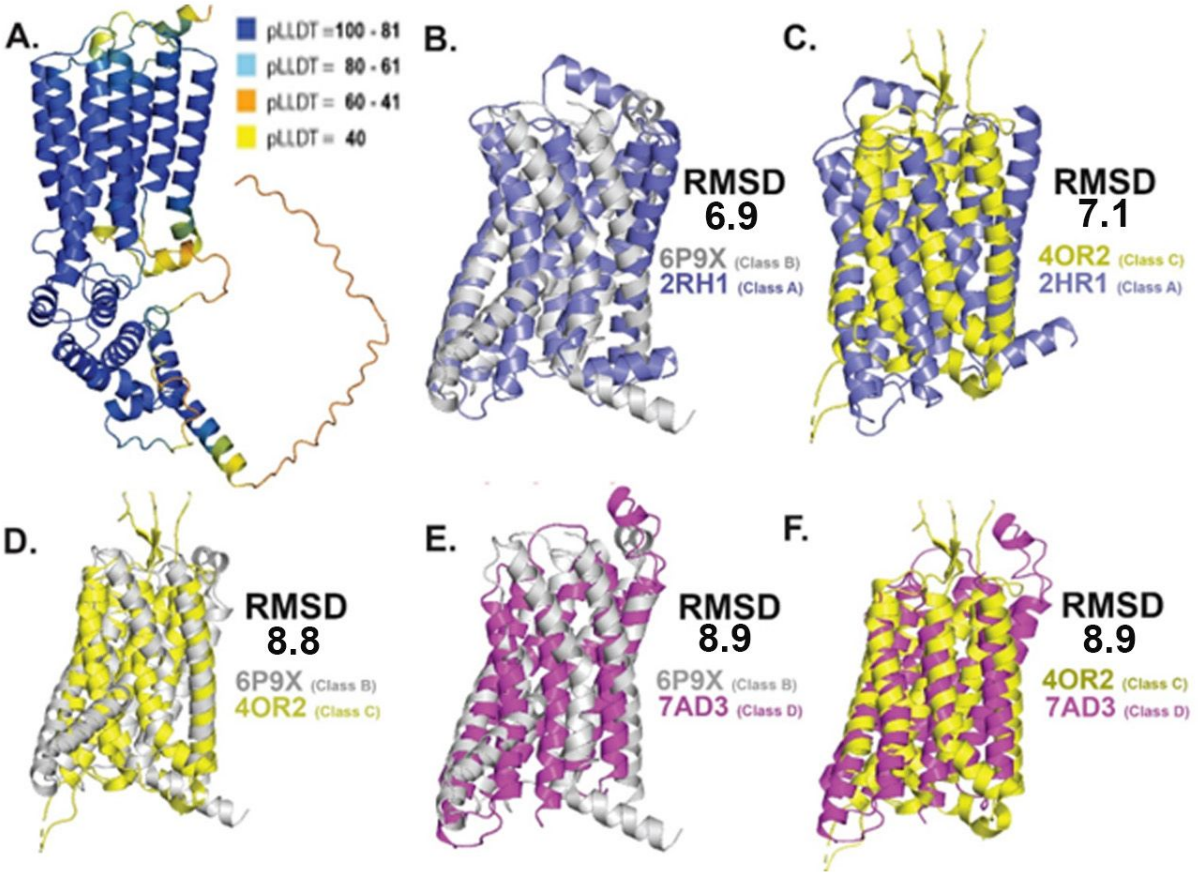

SUPPLEMENTAL INFORMATION

Figures S2-S20 Data from each of the experiments utilizing Microscale Thermophoresis (MST).

The title of each figure represents the data from one MST experiment. For example, the test for the binding affinity between VPS26A and the C-tail of AtRGS1 is "**Supplemental Figure S4"x" VPS26A → RGS1 R-box + C-tail. Supports Figure 2A" and represents a single set of data from which a Kd is determined and used to generate the average Kd seen in the top left cell of Figure 2A.**

	RGS1 R-box + C-tail	RGS1 C-tail	RGS1 pC-tail	RGS1 delta C-tail	RGS1 delta pC-tail	RGS1 C-domain no tail	VPS26A	VPS26B	VPS26AB mutant dimers
VPS26A	Fig. S4	Fig. S2	Fig. S5	n.t.	Fig. S3	Fig. S16	Fig. S18	Fig. S6	Fig. S7
VPS26B	Fig. S14	Fig. S12	Fig. S15	n.t.	Fig. S13	Fig. S17	n.t.	Fig. S19	
VPS26AB dimer	Fig. S11	Fig. S8	Fig. S20	Fig. S9	Fig. S10				

One to four experiments were used to calculate an average Kd +/- StDev. As shown in a table (left) that replicates the table in Figure 2A, the supplemental figure with supporting the Kd in the corresponding cell of Figure 2A is indicated. "n.t." indicates that this target-ligand pair was not tested. As shown in Figure S1-S19, the data are presented in the format of the Monolith NT 115 instrument. The experimental conditions are in the left column. On the right of each experimental output is the calculated binding curve. (top graph) Each point is a value from one MST capillary (different ligand concentration). The fitted curve was obtained from the nanotemper monolith software. The derived parameters shown to the right of the curve are unbound ligand, bound ligand, Kd, response amplitude, and signal to noise ratio. (middle graph) Initial fluorescence for each of the indicated capillaries (different ligand concentrations) with quality controls: sufficient stable signal (average and variation), any detection of ligand adsorption to capillary walls, and ligand-induced fluorescence change. (lower graph) Indication of windows in which data is obtained during change in thermophoresis over the indicated time along with any detection of aggregation or photobleaching.

Supplemental Figure S2A "VPS26A → RGS1 C-tail". Supports Figure 2A

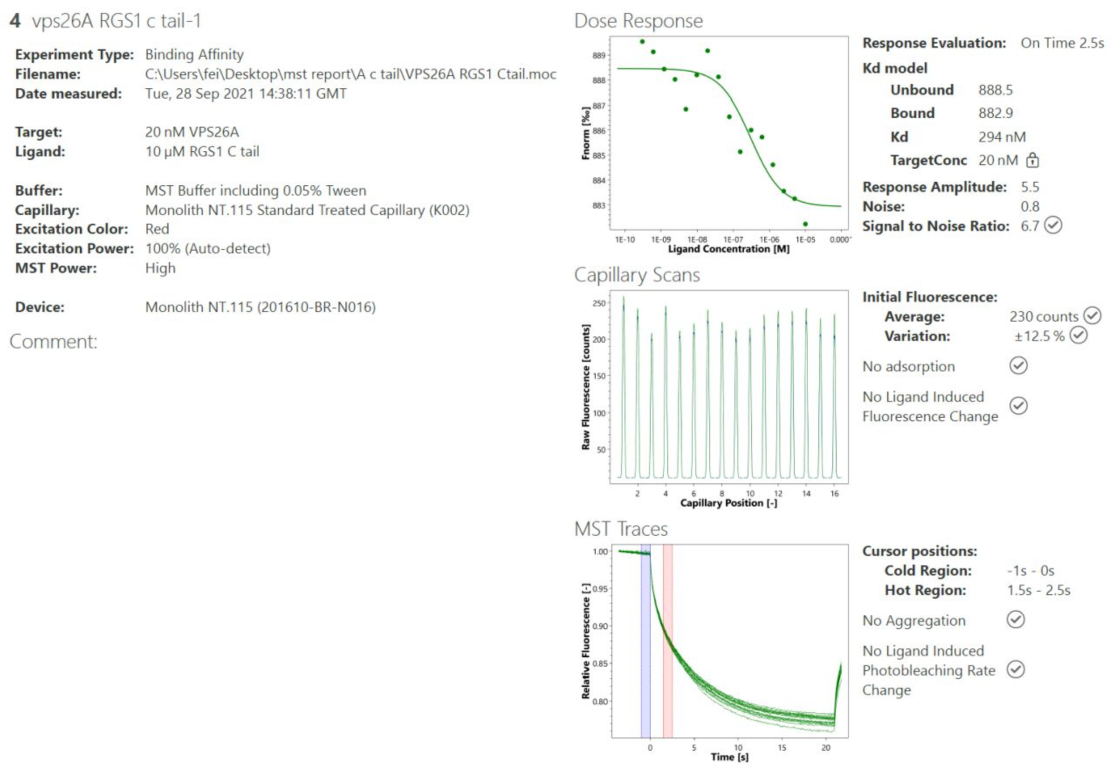

SUPPLEMENTAL INFORMATION

Supplemental Figure S2B "VPS26A → RGS1 C-tail". Supports Figure 2A

5 vps26A RGS1 c tail-2

Experiment Type: Binding Affinity
Filename: C:\Users\fei\Desktop\mst report\A c tail\VPS26A RGS1 Ctail.moc
Date measured: Tue, 28 Sep 2021 15:10:37 GMT

Target: 20 nM VPS26A
Ligand: 90 µM RGS1 C tail

Buffer: MST Buffer including 0.05% Tween
Capillary: Monolith NT.115 Standard Treated Capillary (K002)
Excitation Color: Red
Excitation Power: 100% (Auto-detect)
MST Power: High

Device: Monolith NT.115 (201610-BR-N016)

Comment:

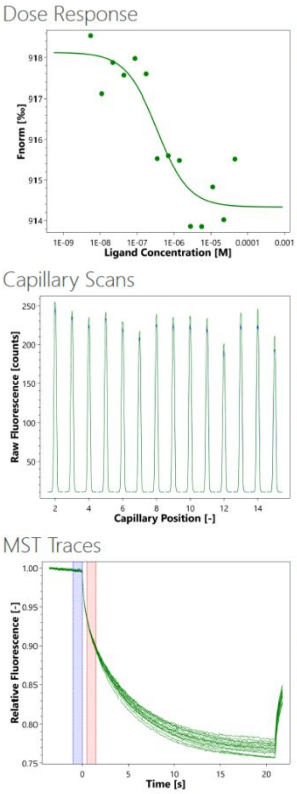

Response Evaluation: On Time 1.5s
Kd model
Unbound 918.1
Bound 914.3
Kd 340 nM
TargetConc 20 nM
Response Amplitude: 3.8
Noise: 0.7
Signal to Noise Ratio: 5.8 ✓

Initial Fluorescence:
Average: 233 counts ✓
Variation: ±13.4 % ✓
No adsorption ✓
No Ligand Induced Fluorescence Change ✓

Cursor positions:
Cold Region: -1s - 0s
Hot Region: 0.5s - 1.5s
No Aggregation ✓
No Ligand Induced Photobleaching Rate Change ✓

Supplemental Figure S2C "VPS26A → RGS1 C-tail". Supports Figure 2A

9 VPS26A RGS1 C tail

Experiment Type: Binding Affinity
Filename: C:\Users\fei\Desktop\mst report\A c tail\VPS26A RGS1 C tail-3.moc
Date measured: Wed, 29 Sep 2021 15:02:31 GMT

Target: 20 nM VPS26A
Ligand: 75 µM RGS1 Ctail

Buffer: MST Buffer including 0.05% Tween
Capillary: Monolith NT.115 Standard Treated Capillary (K002)
Excitation Color: Red
Excitation Power: 100% (Auto-detect)
MST Power: High

Device: Monolith NT.115 (201610-BR-N016)

Comment:

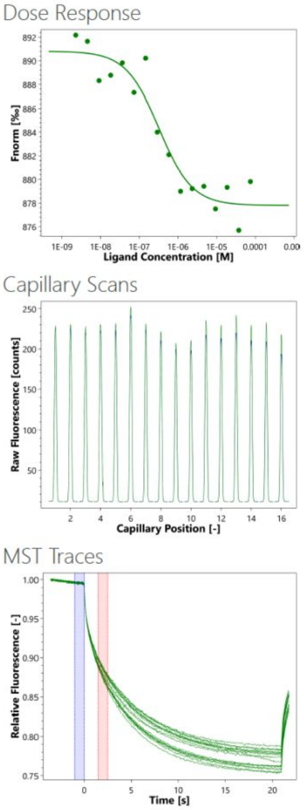

Response Evaluation: On Time 2.5s
Kd model
Unbound 890.8
Bound 877.8
Kd 316 nM
TargetConc 20 nM
Response Amplitude: 13.0
Noise: 1.6
Signal to Noise Ratio: 8.2 ✓

Initial Fluorescence:
Average: 228 counts ✓
Variation: ±10.5 % ✓
No adsorption ✓
No Ligand Induced Fluorescence Change ✓

Cursor positions:
Cold Region: -1s - 0s
Hot Region: 1.5s - 2.5s
No Aggregation ✓
No Ligand Induced Photobleaching Rate Change ✓

SUPPLEMENTAL INFORMATION

Supplemental Figure S3A "VPS26A → RGS1 delta pC-tail". Supports Figure 2A

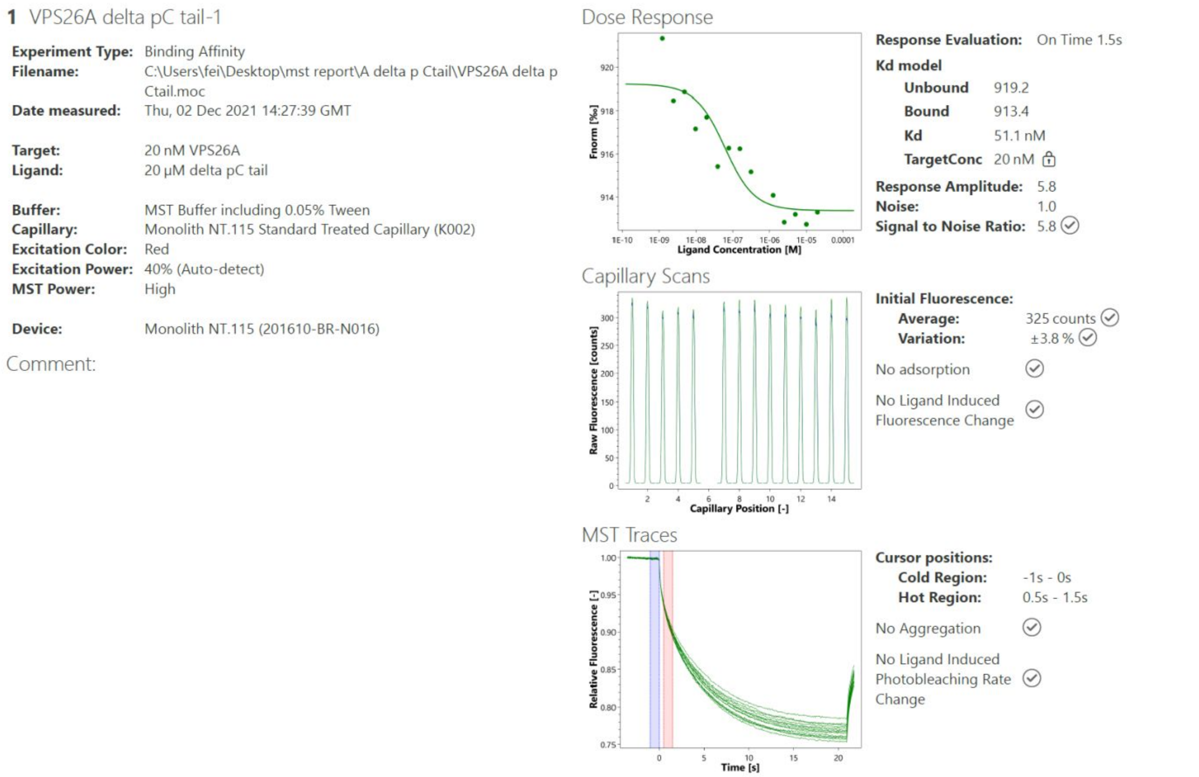

Supplemental Figure S3B "VPS26A → RGS1 delta pC-tail". Supports Figure 2A

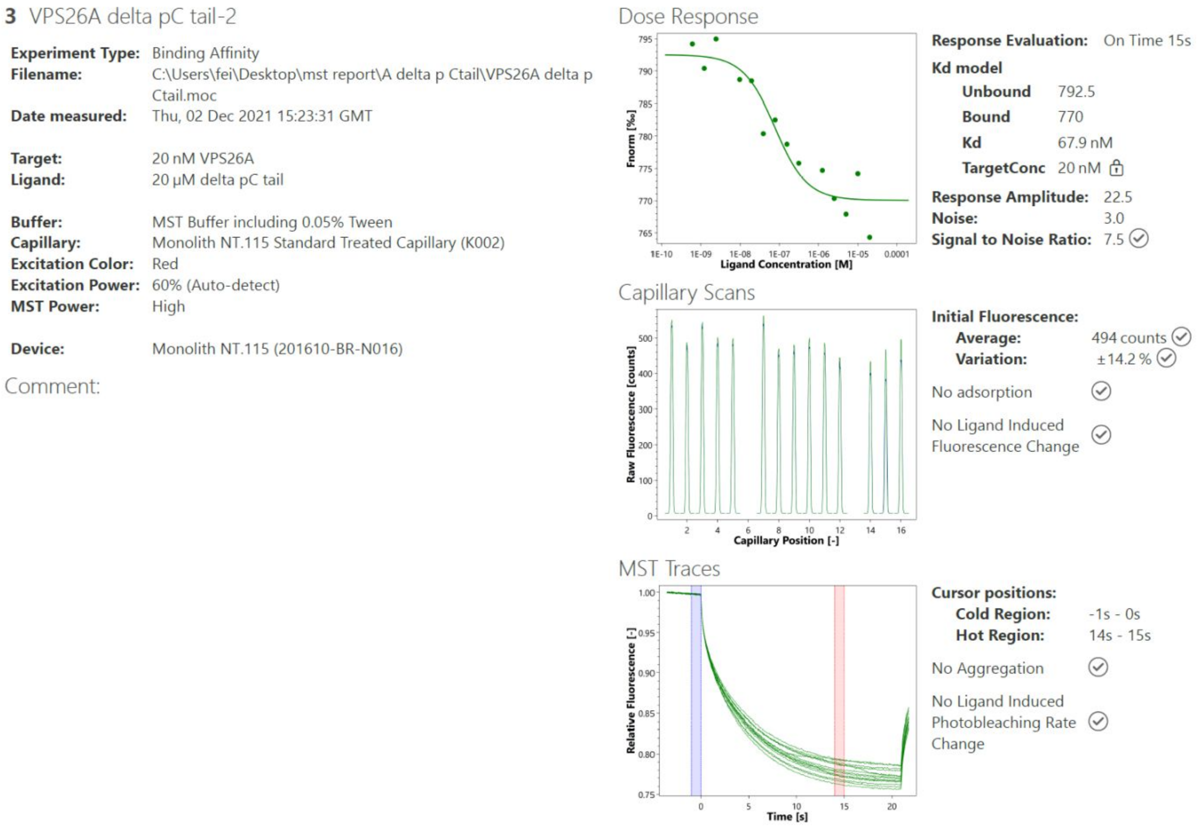

SUPPLEMENTAL INFORMATION

Supplemental Figure S3C "VPS26A → RGS1 delta pC-tail". Supports Figure 2A

4 sb ptcl

Experiment Type: Binding Affinity
Filename: C:\Users\feilou\Desktop\mst report 2023 0822\B delta PCtail\B delta p c tail.moc
Date measured: Thu, 24 Mar 2022 13:21:07 GMT

Target: 20 nM VPS26B
Ligand: 25 μM delta p C tail

Buffer: MST Buffer including 0.05% Tween
Capillary: Monolith NT.115 Standard Treated Capillary (K002)
Excitation Color: Red
Excitation Power: 40% (Auto-detect)
MST Power: High

Device: Monolith NT.115 (201610-BR-N016)

Comment:

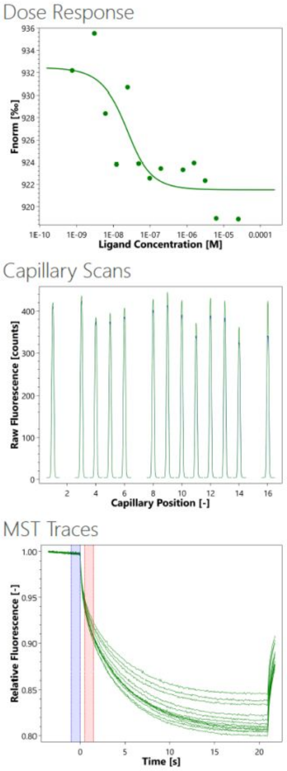

Response Evaluation: On Time 1.5s
Kd model
Unbound 932.4
Bound 921.5
Kd 13.1 nM
TargetConc 20 nM
Response Amplitude: 10.9
Noise: 2.7
Signal to Noise Ratio: 4.0

Initial Fluorescence:
Average: 412 counts
Variation: ±12.2 %
No adsorption
No Ligand Induced Fluorescence Change

Cursor positions:
Cold Region: -1s - 0s
Hot Region: 0.5s - 1.5s
No Aggregation
No Ligand Induced Photobleaching Rate Change

Supplemental Figure S4A "VPS26A → RGS1 R-box + C-tail". Supports Figure 2A

2 sa j5

Experiment Type: Binding Affinity
Filename: C:\Users\feilou\Desktop\jones\fj\fei\sasbj5bmut\j5 bmut.moc
Date measured: Tue, 22 Mar 2022 12:37:39 GMT

Target: 20 nM VPS26A
Ligand: 17.5 μM RGS1 CTD (J5)

Buffer: MST Buffer including 0.05% Tween
Capillary: Monolith NT.115 Standard Treated Capillary (K002)
Excitation Color: Red
Excitation Power: 100% (Auto-detect)
MST Power: High

Device: Monolith NT.115 (201610-BR-N016)

Comment:

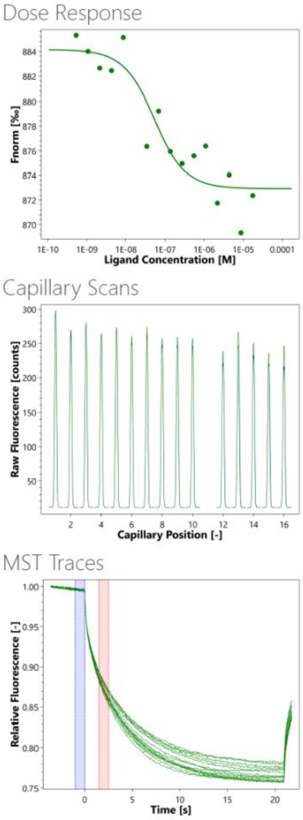

Response Evaluation: On Time 2.5s
Kd model
Unbound 884.2
Bound 872.9
Kd 43.8 nM
TargetConc 20 nM
Response Amplitude: 11.2
Noise: 1.9
Signal to Noise Ratio: 5.8

Initial Fluorescence:
Average: 261 counts
Variation: ±13.6 %
No adsorption
No Ligand Induced Fluorescence Change

Cursor positions:
Cold Region: -1s - 0s
Hot Region: 1.5s - 2.5s
No Aggregation
No Ligand Induced Photobleaching Rate Change

SUPPLEMENTAL INFORMATION

Supplemental Figure S4B "VPS26A → RGS1 R-box + C-tail". Supports Figure 2A

4 sa j52

Experiment Type: Binding Affinity
Filename: C:\Users\feilou\Desktop\jones\fj\fei\sasbj5bmut\j5 bmut.moc
Date measured: Tue, 22 Mar 2022 13:18:28 GMT

Target: 20 nM VPS26A
Ligand: 17.5 μM RGS1 CTD (J5)

Buffer: MST Buffer including 0.05% Tween
Capillary: Monolith NT.115 Standard Treated Capillary (K002)
Excitation Color: Red
Excitation Power: 100% (Auto-detect)
MST Power: High

Device: Monolith NT.115 (201610-BR-N016)

Comment:

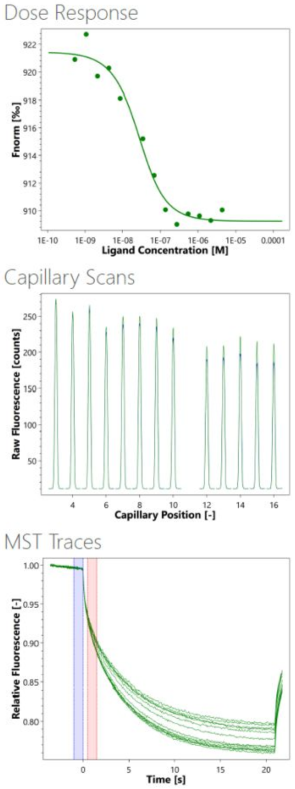

Response Evaluation: On Time 1.5s
Kd model
Unbound 921.4
Bound 909.2
Kd 17.3 nM
TargetConc 20 nM
Response Amplitude: 12.2
Noise: 0.8
Signal to Noise Ratio: 15.5

Initial Fluorescence:
Average: 236 counts
Variation: ±15.7 %
No adsorption
No Ligand Induced Fluorescence Change

Cursor positions:
Cold Region: -1s - 0s
Hot Region: 0.5s - 1.5s
No Aggregation
No Ligand Induced Photobleaching Rate Change

Supplemental Figure S4C "VPS26A → RGS1 R-box + C-tail". Supports Figure 2A

6 sa j5

Experiment Type: Binding Affinity
Filename: C:\Users\feilou\Desktop\jones\fj\fei\sasbj5bmut\j5 bmut.moc
Date measured: Tue, 22 Mar 2022 13:59:06 GMT

Target: 20 nM VPS26A
Ligand: 17.5 μM RGS1 CTD (J5)

Buffer: MST Buffer including 0.05% Tween
Capillary: Monolith NT.115 Standard Treated Capillary (K002)
Excitation Color: Red
Excitation Power: 100% (Auto-detect)
MST Power: High

Device: Monolith NT.115 (201610-BR-N016)

Comment:

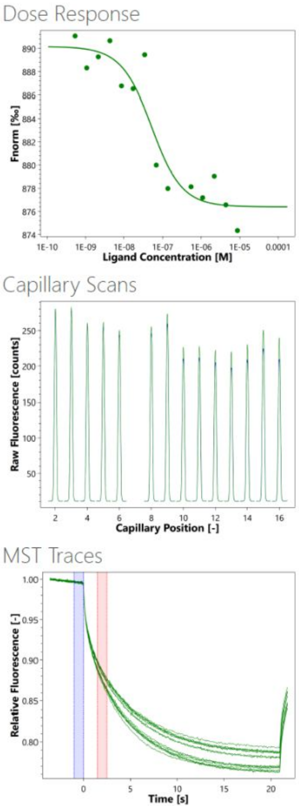

Response Evaluation: On Time 2.5s
Kd model
Unbound 890.2
Bound 876.4
Kd 41.9 nM
TargetConc 20 nM
Response Amplitude: 13.8
Noise: 2.0
Signal to Noise Ratio: 7.0

Initial Fluorescence:
Average: 249 counts
Variation: ±13.4 %
No adsorption
No Ligand Induced Fluorescence Change

Cursor positions:
Cold Region: -1s - 0s
Hot Region: 1.5s - 2.5s
No Aggregation
No Ligand Induced Photobleaching Rate Change

SUPPLEMENTAL INFORMATION

Supplemental Figure S4D "VPS26A → RGS1 R-box + C-tail". Supports Figure 2A

5 Experiment 5

Experiment Type: Binding Affinity
Filename: C:\Users\fei\Desktop\NEW MST DATA\aB J5.moc
Date measured: Wed, 23 Aug 2023 14:27:27 GMT

Target: 20 nM A
Ligand: 20 µM J5

Buffer: MST Buffer including 0.05% Tween
Capillary: Monolith NT.115 Standard Treated Capillary (K002)
Excitation Color: Red
Excitation Power: 80% (Auto-detect)
MST Power: High

Device: Monolith NT.115 (201610-BR-N016)

Comment:

Dose Response

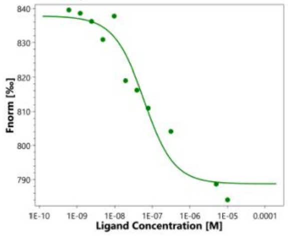

Response Evaluation: On Time 10s

Kd model
Unbound 837.8
Bound 788.7
Kd 50.2 nM
TargetConc 20 nM

Response Amplitude: 49.1
Noise: 4.7
Signal to Noise Ratio: 10.5

Capillary Scans

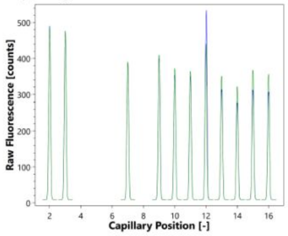

Initial Fluorescence:
Average: 393 counts
Variation: ±22.9 %

No adsorption

No Ligand Induced Fluorescence Change

MST Traces

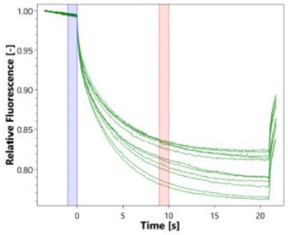

Cursor positions:
Cold Region: -1s - 0s
Hot Region: 9s - 10s

No Aggregation

No Ligand Induced Photobleaching Rate Change

Supplemental Figure S5A "VPS26A → RGS1 pC-tail". Supports Figure 2A

6 Experiment 6

Experiment Type: Binding Affinity
Filename: C:\Users\fei\Desktop\NEW MST DATA\aB J5.moc
Date measured: Wed, 23 Aug 2023 14:58:23 GMT

Target: 20 nM A
Ligand: 50 µM PCTL

Buffer: MST Buffer including 0.05% Tween
Capillary: Monolith NT.115 Standard Treated Capillary (K002)
Excitation Color: Red
Excitation Power: 80% (Auto-detect)
MST Power: Medium

Device: Monolith NT.115 (201610-BR-N016)

Comment:

Dose Response

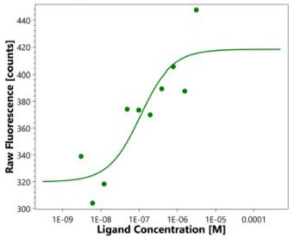

Response Evaluation: Initial Fluorescence

Kd model
Unbound 319.8
Bound 418.2
Kd 98.1 nM
TargetConc 20 nM

Response Amplitude: 98.4
Noise: 19.4
Signal to Noise Ratio: 5.1

Capillary Scans

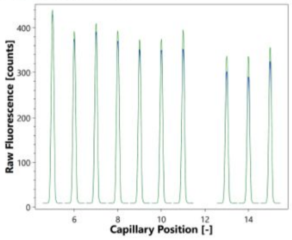

Initial Fluorescence:
Average: 380 counts
Variation: ±15.5 %

No adsorption

Ligand Induced Fluorescence Change
Binding-specific change

MST Traces

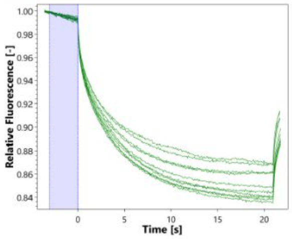

Cursor positions:
Cold Region: -3s - 0s
Hot Region: N/A

No Aggregation

No Ligand Induced Photobleaching Rate Change

SUPPLEMENTAL INFORMATION

Supplemental Figure S5B "VPS26A → RGS1 pC-tail". Supports Figure 2A

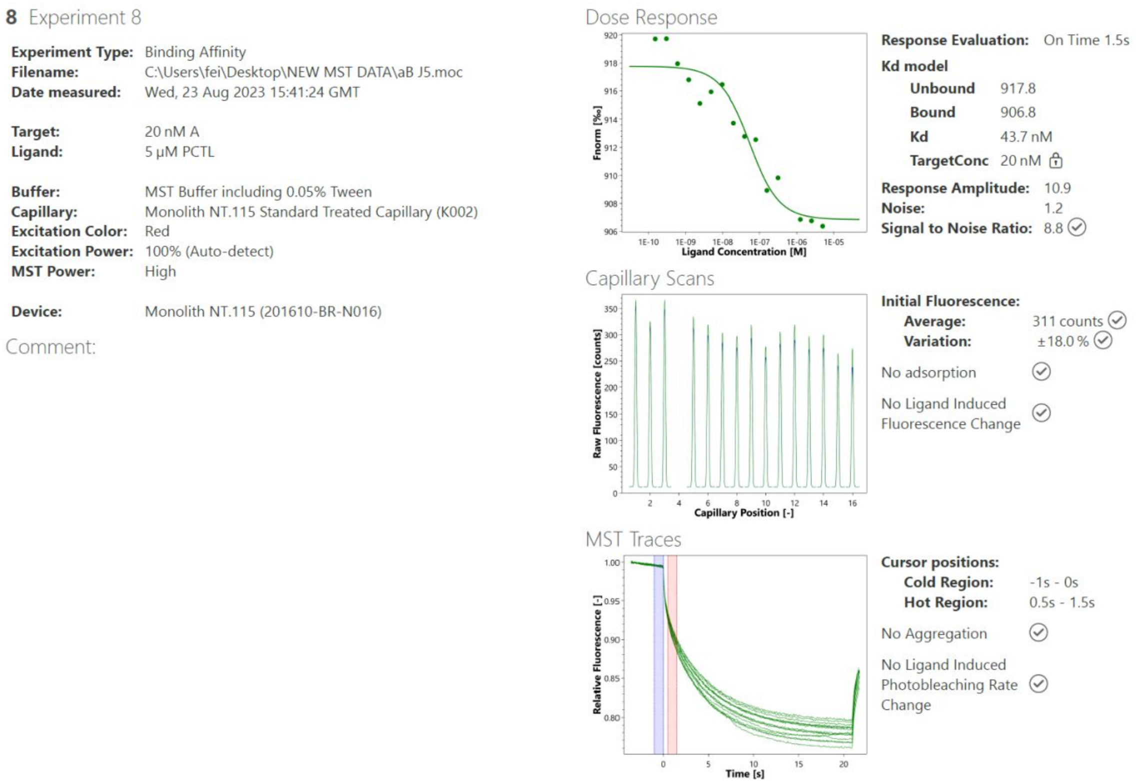

Supplemental Figure S6A "VPS26A → VPS26B". Supports Figure 2A

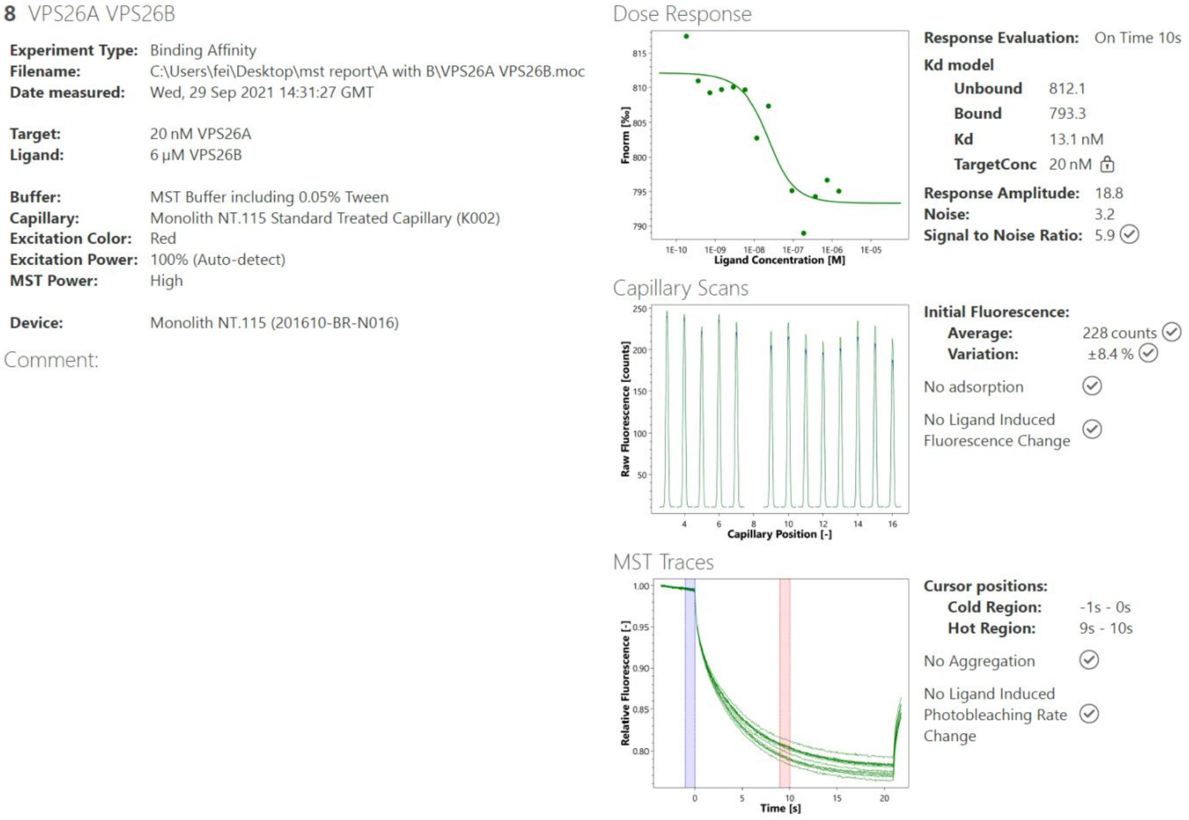

SUPPLEMENTAL INFORMATION

Supplemental Figure S6B "VPS26A → VPS26B". Supports Figure 2A

5 VPS26A VPS26B

Experiment Type: Binding Affinity
Filename: C:\Users\fei\Desktop\mst report\A with B\VPS26A VPS26B 2.moc
Date measured: Wed, 22 Sep 2021 13:35:16 GMT

Target: 20 nM VPS26A
Ligand: 7.5 µM VPS26B

Buffer: MST Buffer including 0.05% Tween
Capillary: Monolith NT.115 Standard Treated Capillary (K002)
Excitation Color: Red
Excitation Power: 100% (Auto-detect)
MST Power: High

Device: Monolith NT.115 (201610-BR-N016)

Comment:

Dose Response

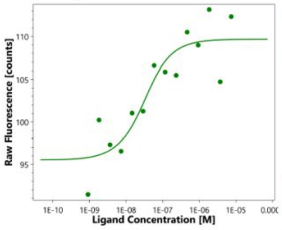

Response Evaluation: Initial Fluorescence

Kd model
Unbound 95.5
Bound 109.7
Kd 22.6 nM
TargetConc 20 nM

Response Amplitude: 14.2
Noise: 2.8
Signal to Noise Ratio: 5.1

Capillary Scans

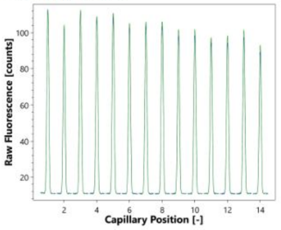

Initial Fluorescence:
Average: 104 counts
Variation: ±11.1 %

No adsorption

Ligand Induced Fluorescence Change

Binding-specific change

MST Traces

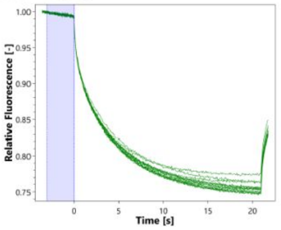

Cursor positions:
Cold Region: -3s - 0s
Hot Region: N/A

No Aggregation

No Ligand Induced Photobleaching Rate Change

Supplemental Figure S7A "VPS26AB mutant dimers". Supports Figure 2A

3 sa sbmut

Experiment Type: Binding Affinity
Filename: C:\Users\feilou\Desktop\mst report\A with B interface mutation\A with B interface muation.moc
Date measured: Tue, 22 Mar 2022 12:57:57 GMT

Target: 20 nM VPS26A
Ligand: 6 µM VPS26B interface mutaion

Buffer: MST Buffer including 0.05% Tween
Capillary: Monolith NT.115 Standard Treated Capillary (K002)
Excitation Color: Red
Excitation Power: 100% (Auto-detect)
MST Power: High

Device: Monolith NT.115 (201610-BR-N016)

Comment:

Dose Response

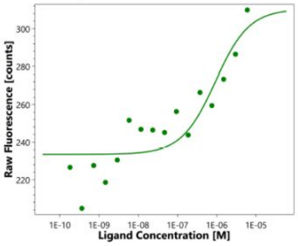

Response Evaluation: Initial Fluorescence

Kd model
Unbound 233.5
Bound 310.4
Kd 908 nM
TargetConc 20 nM

Response Amplitude: 76.9
Noise: 12.7
Signal to Noise Ratio: 6.0

Capillary Scans

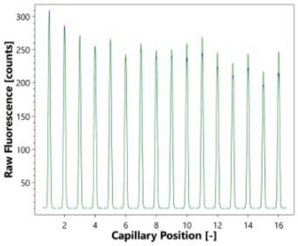

Initial Fluorescence:
Average: 255 counts
Variation: ±18.8 %

No adsorption

Ligand Induced Fluorescence Change

Binding-specific change

MST Traces

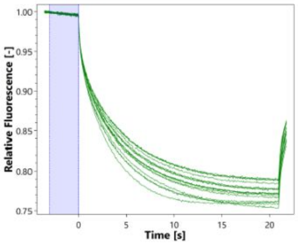

Cursor positions:
Cold Region: -3s - 0s
Hot Region: N/A

No Aggregation

No Ligand Induced Photobleaching Rate Change

SUPPLEMENTAL INFORMATION

Supplemental Figure S7B "VPS26AB mutant dimers". Supports Figure 2A

5 sa sbmut

Experiment Type: Binding Affinity
Filename: C:\Users\feilou\Desktop\mst report\A with B interface mutation\A with B interface muation.moc
Date measured: Tue, 22 Mar 2022 13:39:06 GMT

Target: 20 nM VPS26A
Ligand: 6 µM VPS26B interface mutaion

Buffer: MST Buffer including 0.05% Tween
Capillary: Monolith NT.115 Standard Treated Capillary (K002)
Excitation Color: Red
Excitation Power: 100% (Auto-detect)
MST Power: High

Device: Monolith NT.115 (201610-BR-N016)

Comment:

Dose Response

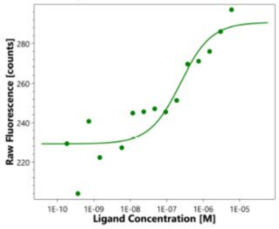

Response Evaluation: Initial Fluorescence

Kd model
Unbound 229.2
Bound 290.8
Kd 228 nM
TargetConc 20 nM

Response Amplitude: 61.5
Noise: 9.9
Signal to Noise Ratio: 6.2 ✓

Capillary Scans

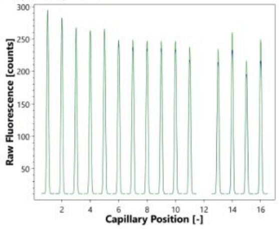

Initial Fluorescence:
Average: 253 counts ✓
Variation: ±14.9 % !

No adsorption ✓

Ligand Induced Fluorescence Change !
Binding-specific change ✓

MST Traces

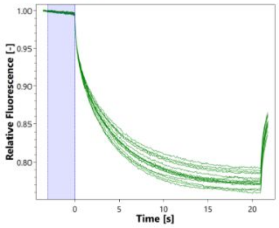

Cursor positions:
Cold Region: -3s - 0s
Hot Region: N/A

No Aggregation ✓

No Ligand Induced Photobleaching Rate Change ✓

Supplemental Figure S7C "VPS26AB mutant dimers". Supports Figure 2A

8 sa sbmut

Experiment Type: Binding Affinity
Filename: C:\Users\feilou\Desktop\mst report\A with B interface mutation\A with B interface muation.moc
Date measured: Tue, 22 Mar 2022 14:19:16 GMT

Target: 20 nM VPS26A
Ligand: 6 µM VPS26B interface mutaion

Buffer: MST Buffer including 0.05% Tween
Capillary: Monolith NT.115 Standard Treated Capillary (K002)
Excitation Color: Red
Excitation Power: 100% (Auto-detect)
MST Power: High

Device: Monolith NT.115 (201610-BR-N016)

Comment:

Dose Response

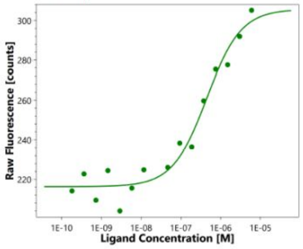

Response Evaluation: Initial Fluorescence

Kd model
Unbound 216.3
Bound 305.6
Kd 439 nM
TargetConc 20 nM

Response Amplitude: 89.3
Noise: 6.3
Signal to Noise Ratio: 14.1 ✓

Capillary Scans

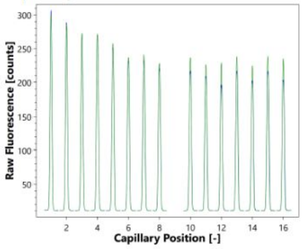

Initial Fluorescence:
Average: 248 counts ✓
Variation: ±20.8 % ✓

No adsorption ✓

Ligand Induced Fluorescence Change !
Binding-specific change ✓

MST Traces

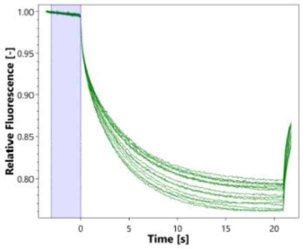

Cursor positions:
Cold Region: -3s - 0s
Hot Region: N/A

No Aggregation ✓

No Ligand Induced Photobleaching Rate Change ✓

SUPPLEMENTAL INFORMATION

Supplemental Figure S8A "VPS26AB → RGS1 C-tail". Supports Figure 2A

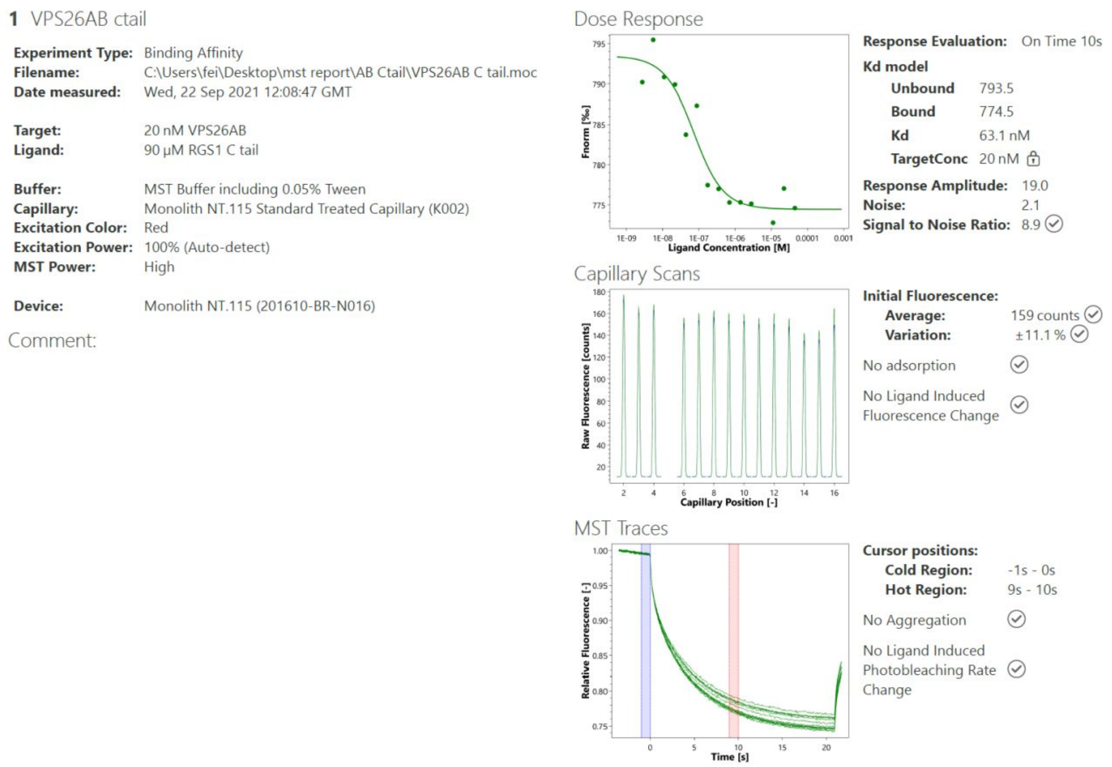

Supplemental Figure S8B "VPS26AB → RGS1 C-tail". Supports Figure 2A

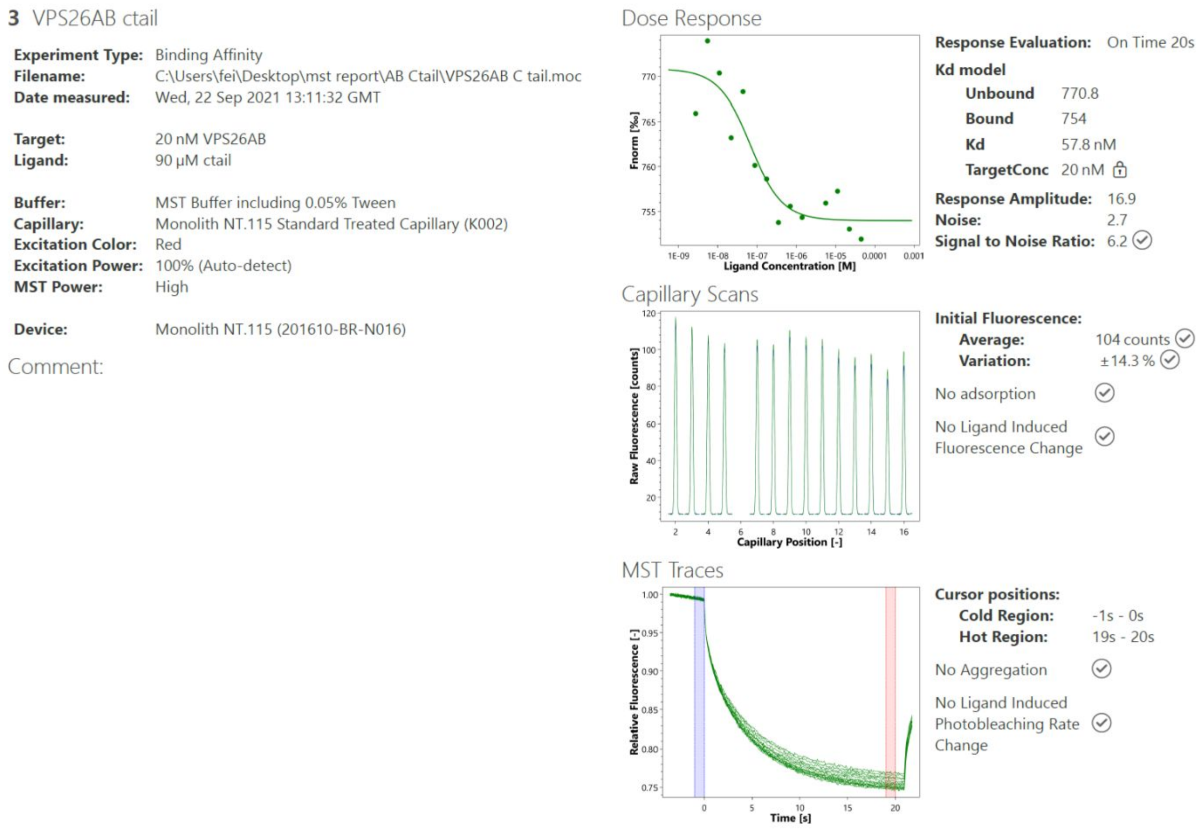

SUPPLEMENTAL INFORMATION

Supplemental Figure S8C "VPS26AB → RGS1 C-tail". Supports Figure 2A

10 VPS26AB RGS1 Ctail

Experiment Type: Binding Affinity
Filename: C:\Users\fei\Desktop\mst report\A c tail\VPS26A RGS1 C tail-3.moc
Date measured: Wed, 29 Sep 2021 15:32:10 GMT

Target: 20 nM VPS26AB
Ligand: 75 µM RGS1 Ctail

Buffer: MST Buffer including 0.05% Tween
Capillary: Monolith NT.115 Standard Treated Capillary (K002)
Excitation Color: Red
Excitation Power: 100% (Auto-detect)
MST Power: High

Device: Monolith NT.115 (201610-BR-N016)

Comment:

Dose Response

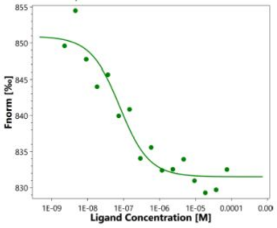

Response Evaluation: On Time 5s
Kd model
Unbound 850.9
Bound 831.5
Kd 68.7 nM
TargetConc 20 nM
Response Amplitude: 19.4
Noise: 2.1
Signal to Noise Ratio: 9.2

Capillary Scans

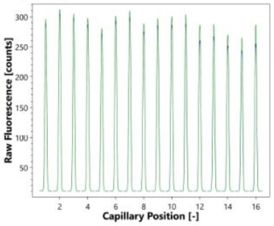

Initial Fluorescence:
Average: 293 counts
Variation: ±9.5 %
No adsorption
No Ligand Induced Fluorescence Change

MST Traces

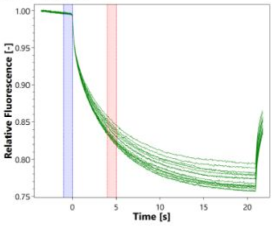

Cursor positions:
Cold Region: -1s - 0s
Hot Region: 4s - 5s
No Aggregation
No Ligand Induced Photobleaching Rate Change

Supplemental Figure S9A "VPS26AB → RGS1 delta C-tail". Supports Figure 2A

1 ab Delta ctl1

Experiment Type: Binding Affinity
Filename: C:\Users\fei\Desktop\mst report 2023 0822\AB delta c tail\ab tl.moc
Date measured: Fri, 11 Aug 2023 13:50:13 GMT

Target: 20 nM ab
Ligand: 30 µM tctl

Buffer: MST Buffer including 0.05% Tween
Capillary: Monolith NT.115 Standard Treated Capillary (K002)
Excitation Color: Red
Excitation Power: 100% (Auto-detect)
MST Power: Medium

Device: Monolith NT.115 (201610-BR-N016)

Comment:

Dose Response

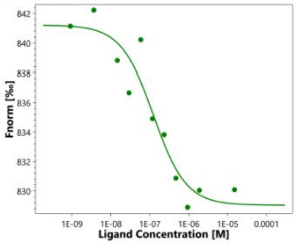

Response Evaluation: On Time 15s
Kd model
Unbound 841.2
Bound 829
Kd 115 nM
TargetConc 20 nM
Response Amplitude: 12.2
Noise: 1.4
Signal to Noise Ratio: 8.5

Capillary Scans

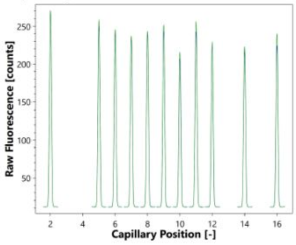

Initial Fluorescence:
Average: 242 counts
Variation: ±12.0 %
No adsorption
No Ligand Induced Fluorescence Change

MST Traces

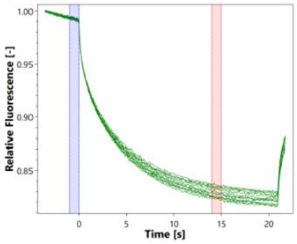

Cursor positions:
Cold Region: -1s - 0s
Hot Region: 14s - 15s
No Aggregation
No Ligand Induced Photobleaching Rate Change

SUPPLEMENTAL INFORMATION

Supplemental Figure S9B "VPS26AB → RGS1 delta C-tail". Supports Figure 2A

2 ab Delta ctl2

Experiment Type: Binding Affinity
Filename: C:\Users\fei\Desktop\mst report 2023 0822\AB delta c tail\ab tl.moc
Date measured: Fri, 11 Aug 2023 14:16:58 GMT

Target: 20 nM ab
Ligand: 30 µM tctl

Buffer: MST Buffer including 0.05% Tween
Capillary: Monolith NT.115 Standard Treated Capillary (K002)
Excitation Color: Red
Excitation Power: 100% (Auto-detect)
MST Power: Medium

Device: Monolith NT.115 (201610-BR-N016)

Comment:

Dose Response

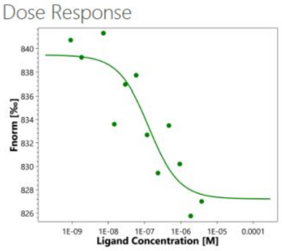

Response Evaluation: On Time 15s
Kd model
Unbound 839.4
Bound 827.2
Kd 121 nM
TargetConc 20 nM
Response Amplitude: 12.2
Noise: 2.3
Signal to Noise Ratio: 5.2

Capillary Scans

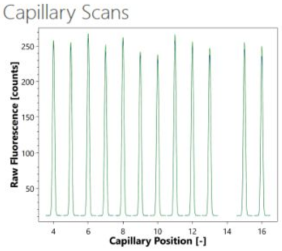

Initial Fluorescence:
Average: 254 counts
Variation: ±6.3 %
No adsorption
No Ligand Induced Fluorescence Change

MST Traces

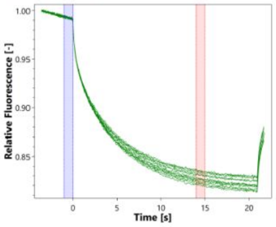

Cursor positions:
Cold Region: -1s - 0s
Hot Region: 14s - 15s
No Aggregation
No Ligand Induced Photobleaching Rate Change

Supplemental Figure S9C "VPS26AB → RGS1 delta C-tail". Supports Figure 2A

3 ab Delta ctl3

Experiment Type: Binding Affinity
Filename: C:\Users\fei\Desktop\mst report 2023 0822\AB delta c tail\ab tl.moc
Date measured: Fri, 11 Aug 2023 14:37:24 GMT

Target: 20 nM ab
Ligand: 30 µM tctl

Buffer: MST Buffer including 0.05% Tween
Capillary: Monolith NT.115 Standard Treated Capillary (K002)
Excitation Color: Red
Excitation Power: 100% (Auto-detect)
MST Power: Medium

Device: Monolith NT.115 (201610-BR-N016)

Comment:

Dose Response

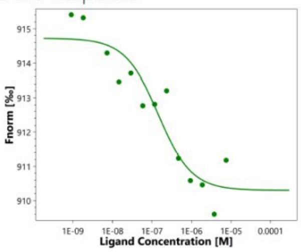

Response Evaluation: On Time 2.5s
Kd model
Unbound 914.7
Bound 910.3
Kd 135 nM
TargetConc 20 nM
Response Amplitude: 4.4
Noise: 0.7
Signal to Noise Ratio: 6.6

Capillary Scans

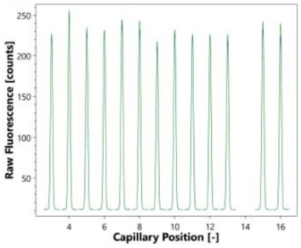

Initial Fluorescence:
Average: 234 counts
Variation: ±8.9 %
No adsorption
No Ligand Induced Fluorescence Change

MST Traces

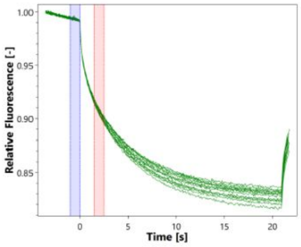

Cursor positions:
Cold Region: -1s - 0s
Hot Region: 1.5s - 2.5s
No Aggregation
No Ligand Induced Photobleaching Rate Change

SUPPLEMENTAL INFORMATION

Supplemental Figure S10A “VPS26AB → RGS1 delta pC-tail”. Supports Figure 2A

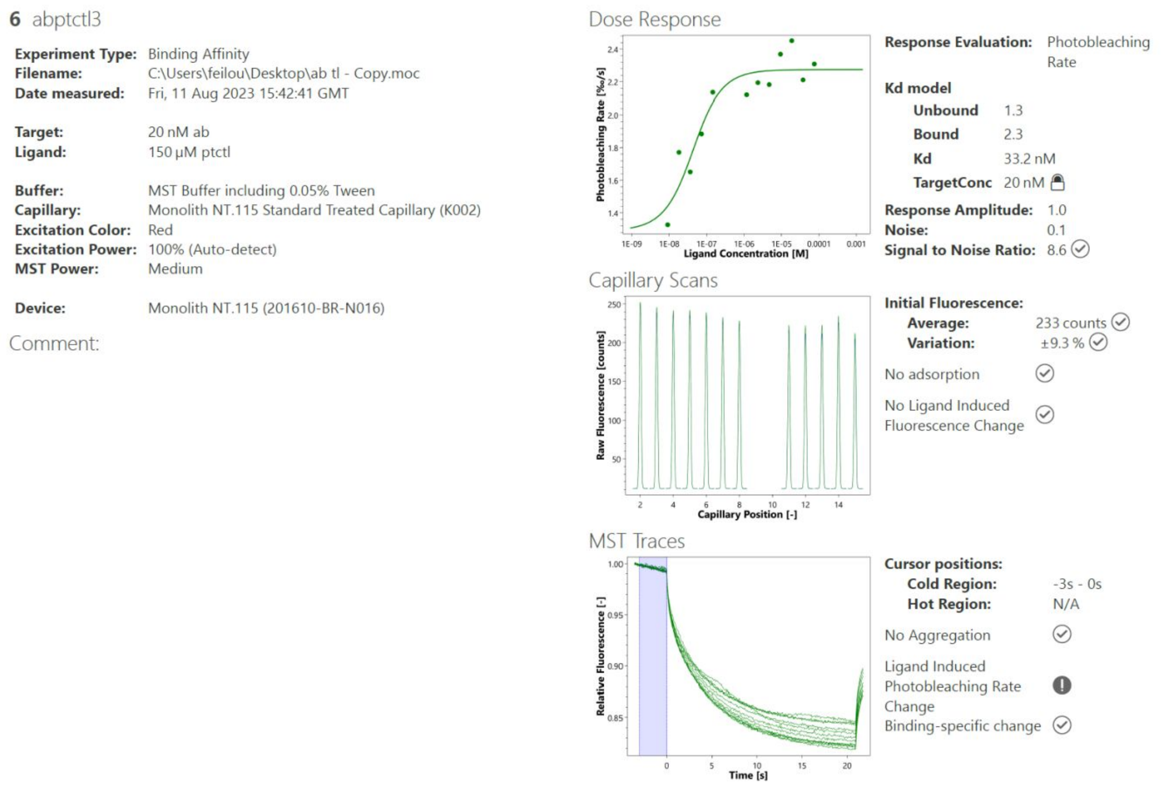

Supplemental Figure S10B “VPS26AB → RGS1 delta pC-tail”. Supports Figure 2A

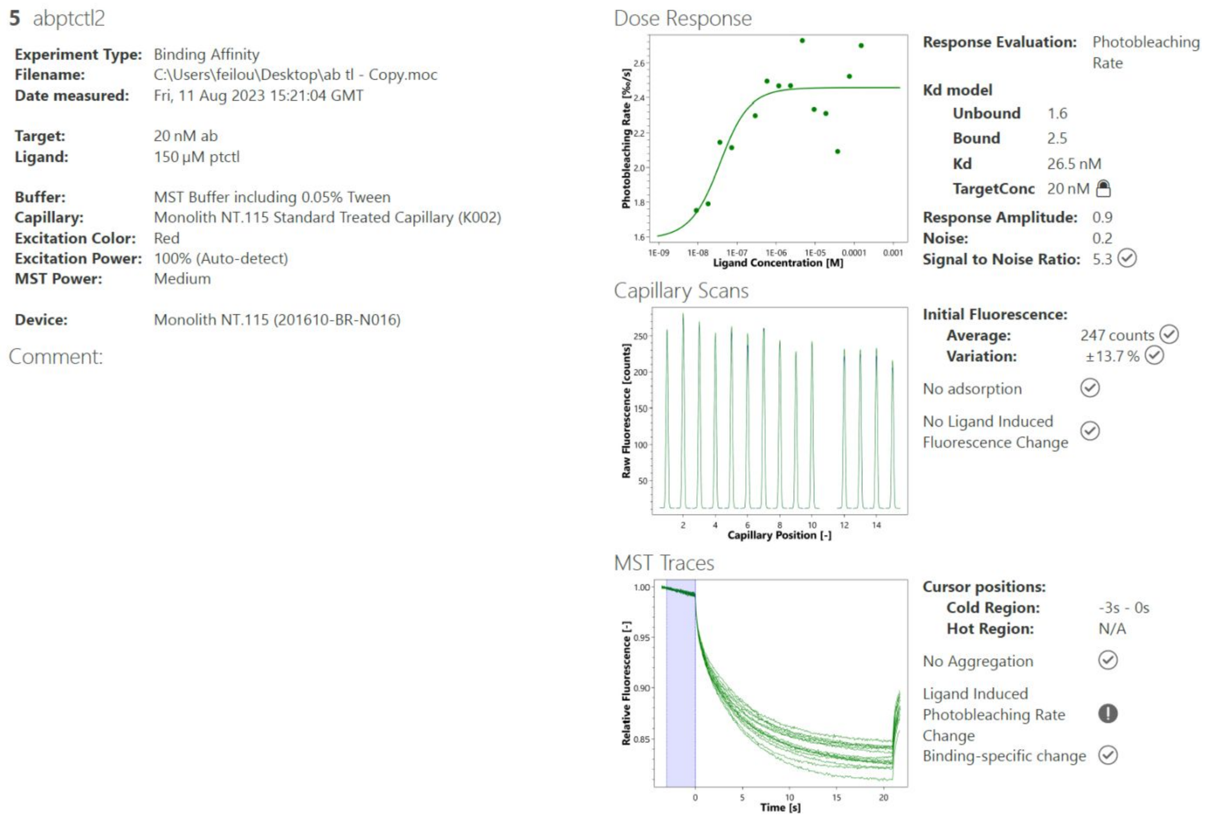

SUPPLEMENTAL INFORMATION

Supplemental Figure S10C “VPS26AB → RGS1 delta pC-tail”. Supports Figure 2A

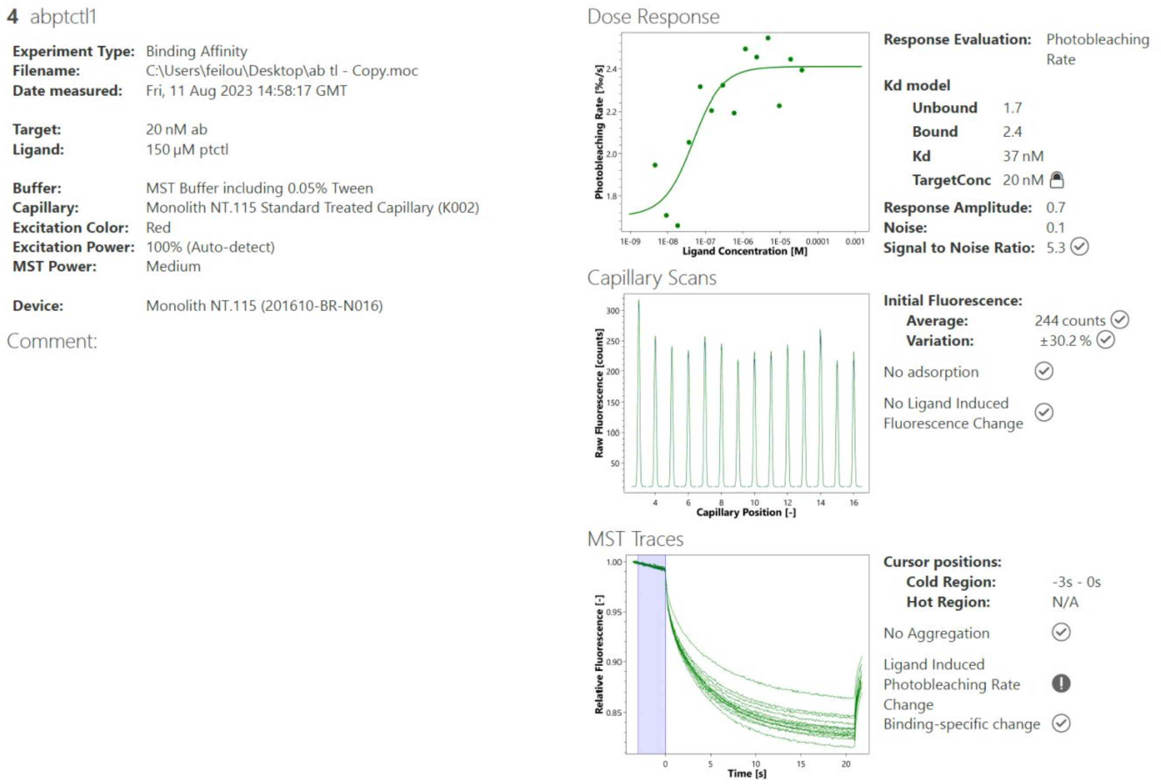

Supplemental Figure S11A “VPS26AB → RGS1 R-box +C-tail”. Supports Figure 2A

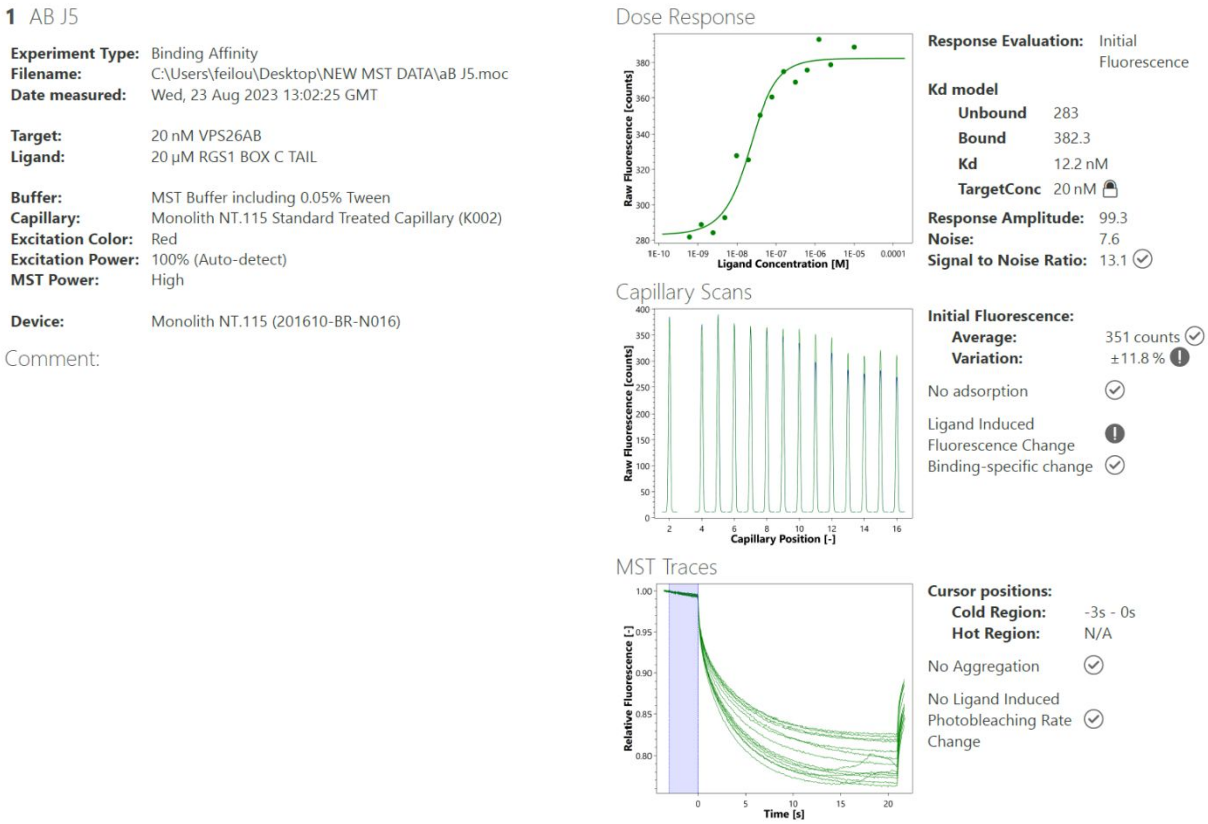

SUPPLEMENTAL INFORMATION

Supplemental Figure S11B "VPS26AB → RGS1 R-box +C-tail". Supports Figure 2A

2 AB J5 2

Experiment Type: Binding Affinity
Filename: C:\Users\fei\Desktop\NEW MST DATA\aB J5.moc
Date measured: Wed, 23 Aug 2023 13:24:25 GMT

Target: 20 nM VPS26AB
Ligand: 20 µM J5

Buffer: MST Buffer including 0.05% Tween
Capillary: Monolith NT.115 Standard Treated Capillary (K002)
Excitation Color: Red
Excitation Power: 100% (Auto-detect)
MST Power: High

Device: Monolith NT.115 (201610-BR-N016)

Comment:

Dose Response

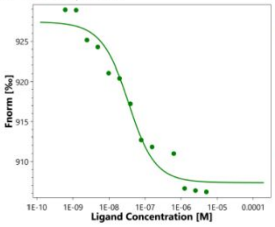

Response Evaluation: On Time 1.5s
Kd model
Unbound 927.4
Bound 907.3
Kd 24.5 nM
TargetConc 20 nM
Response Amplitude: 20.1
Noise: 1.6
Signal to Noise Ratio: 12.5

Capillary Scans

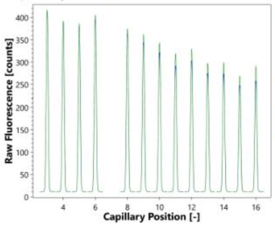

Initial Fluorescence:
Average: 345 counts
Variation: ±21.9 %
No adsorption
No Ligand Induced Fluorescence Change

MST Traces

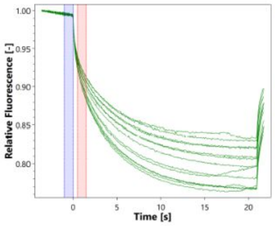

Cursor positions:
Cold Region: -1s - 0s
Hot Region: 0.5s - 1.5s
No Aggregation
No Ligand Induced Photobleaching Rate Change

Supplemental Figure S11C "VPS26AB → RGS1 R-box +C-tail". Supports Figure 2A

9 Experiment 9

Experiment Type: Binding Affinity
Filename: C:\Users\fei\Desktop\NEW MST DATA\aB J5.moc
Date measured: Wed, 23 Aug 2023 16:02:12 GMT

Target: 20 nM AB
Ligand: 20 µM J5

Buffer: MST Buffer including 0.05% Tween
Capillary: Monolith NT.115 Standard Treated Capillary (K002)
Excitation Color: Red
Excitation Power: 100% (Auto-detect)
MST Power: Medium

Device: Monolith NT.115 (201610-BR-N016)

Comment:

Dose Response

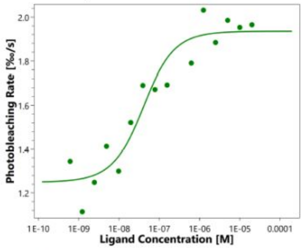

Response Evaluation: Photobleaching Rate
Kd model
Unbound 1.2
Bound 1.9
Kd 30.3 nM
TargetConc 20 nM
Response Amplitude: 0.7
Noise: 0.1
Signal to Noise Ratio: 7.8

Capillary Scans

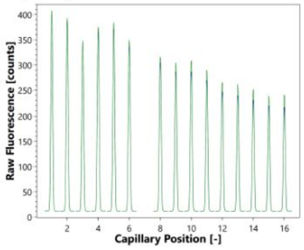

Initial Fluorescence:
Average: 316 counts
Variation: ±29.3 %
No adsorption
No Ligand Induced Fluorescence Change

MST Traces

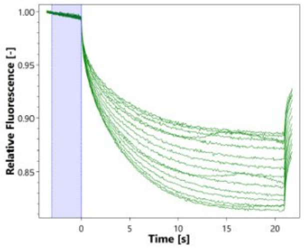

Cursor positions:
Cold Region: -3s - 0s
Hot Region: N/A
No Aggregation
No Ligand Induced Photobleaching Rate Change

SUPPLEMENTAL INFORMATION

Supplemental Figure S12A "VPS26B → RGS1 C-tail". Supports Figure 2A

5 B CTL-1

Experiment Type: Binding Affinity
Filename: C:\Users\fei\Desktop\mst report\B ctail\VPS26B RGS1 C tail.moc
Date measured: Wed, 29 Sep 2021 13:03:53 GMT

Target: 20 nM VPS26B
Ligand: 10 µM RGS1 Ctail

Buffer: MST Buffer including 0.05% Tween
Capillary: Monolith NT.115 Standard Treated Capillary (K002)
Excitation Color: Red
Excitation Power: 100% (Auto-detect)
MST Power: High

Device: Monolith NT.115 (201610-BR-N016)

Comment:

Dose Response

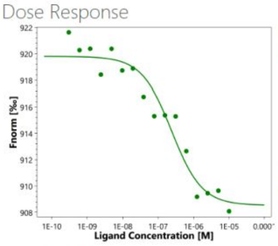

Response Evaluation: On Time 1.5s
Kd model
Unbound 919.8
Bound 908.5
Kd 232 nM
TargetConc 20 nM
Response Amplitude: 11.3
Noise: 1.1
Signal to Noise Ratio: 10.1

Capillary Scans

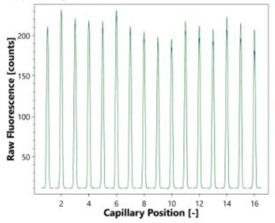

Initial Fluorescence:
Average: 214 counts
Variation: ±8.6 %
No adsorption
No Ligand Induced Fluorescence Change

MST Traces

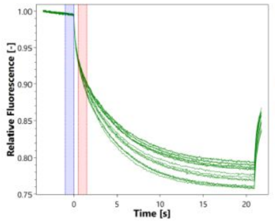

Cursor positions:
Cold Region: -1s - 0s
Hot Region: 0.5s - 1.5s
No Aggregation
No Ligand Induced Photobleaching Rate Change

Supplemental Figure S12B "VPS26B → RGS1 C-tail". Supports Figure 2A

6 B ctl-2

Experiment Type: Binding Affinity
Filename: C:\Users\fei\Desktop\mst report\B ctail\VPS26B RGS1 C tail.moc
Date measured: Wed, 29 Sep 2021 13:31:36 GMT

Target: 20 nM VPS26B
Ligand: 10 µM RGS1 Ctail

Buffer: MST Buffer including 0.05% Tween
Capillary: Monolith NT.115 Standard Treated Capillary (K002)
Excitation Color: Red
Excitation Power: 100% (Auto-detect)
MST Power: High

Device: Monolith NT.115 (201610-BR-N016)

Comment:

Dose Response

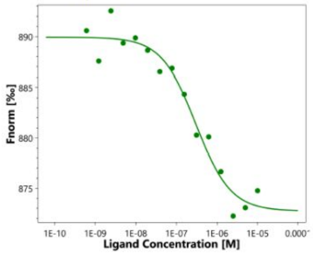

Response Evaluation: On Time 2.5s
Kd model
Unbound 889.9
Bound 872.7
Kd 295 nM
TargetConc 20 nM
Response Amplitude: 17.2
Noise: 1.4
Signal to Noise Ratio: 12.0

Capillary Scans

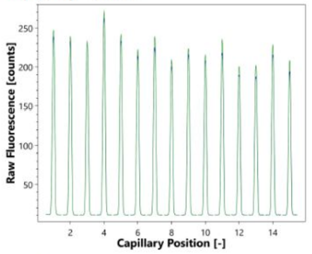

Initial Fluorescence:
Average: 227 counts
Variation: ±18.6 %
No adsorption
No Ligand Induced Fluorescence Change

MST Traces

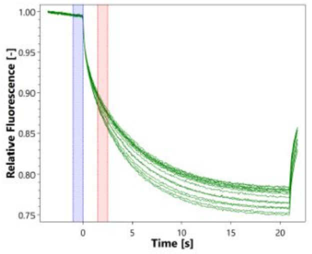

Cursor positions:
Cold Region: -1s - 0s
Hot Region: 1.5s - 2.5s
No Aggregation
No Ligand Induced Photobleaching Rate Change

SUPPLEMENTAL INFORMATION

Supplemental Figure S12C “VPS26B → RGS1 C-tail”. Supports Figure 2A

7 B c tail-3

Experiment Type: Binding Affinity
Filename: C:\Users\fei\Desktop\mst report\B ctail\VPS26B RGS1 C tail.moc
Date measured: Wed, 29 Sep 2021 14:00:56 GMT

Target: 20 nM VPS26B
Ligand: 10 µM RGS1 Ctail

Buffer: MST Buffer including 0.05% Tween
Capillary: Monolith NT.115 Standard Treated Capillary (K002)
Excitation Color: Red
Excitation Power: 100% (Auto-detect)
MST Power: High

Device: Monolith NT.115 (201610-BR-N016)

Comment:

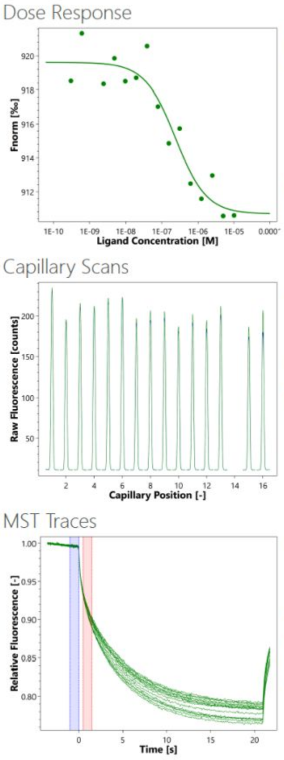

Response Evaluation: On Time 1.5s
Kd model
Unbound 919.6
Bound 910.7
Kd 232 nM
TargetConc 20 nM
Response Amplitude: 8.9
Noise: 1.1
Signal to Noise Ratio: 8.0

Initial Fluorescence:
Average: 206 counts
Variation: ±14.0 %
No adsorption
No Ligand Induced Fluorescence Change

Cursor positions:
Cold Region: -1s - 0s
Hot Region: 0.5s - 1.5s
No Aggregation
No Ligand Induced Photobleaching Rate Change

Supplemental Figure S13A “VPS26B → RGS1 delta pC-tail”. Supports Figure 2A

9 vps26b delta p ctail

Experiment Type: Binding Affinity
Filename: C:\Users\feilou\Desktop\mst report 2023 0822\B delta PCtail\B delta p c tail.moc
Date measured: Thu, 24 Mar 2022 15:20:09 GMT

Target: 20 nM VPS26B
Ligand: 2.5 µM RGS1 delta p c tail

Buffer: MST Buffer including 0.05% Tween
Capillary: Monolith NT.115 Standard Treated Capillary (K002)
Excitation Color: Red
Excitation Power: 40% (Auto-detect)
MST Power: High

Device: Monolith NT.115 (201610-BR-N016)

Comment:

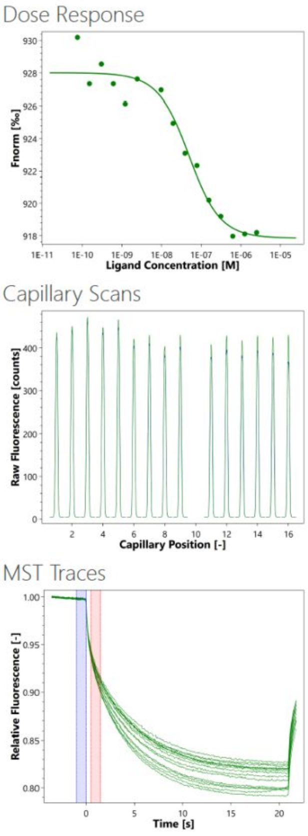

Response Evaluation: On Time 1.5s
Kd model
Unbound 928
Bound 917.9
Kd 39.7 nM
TargetConc 20 nM
Response Amplitude: 10.2
Noise: 0.8
Signal to Noise Ratio: 12.0

Initial Fluorescence:
Average: 433 counts
Variation: ±9.1 %
No adsorption
No Ligand Induced Fluorescence Change

Cursor positions:
Cold Region: -1s - 0s
Hot Region: 0.5s - 1.5s
No Aggregation
No Ligand Induced Photobleaching Rate Change

SUPPLEMENTAL INFORMATION

Supplemental Figure S13B “VPS26B → RGS1 delta pC-tail”. Supports Figure 2A

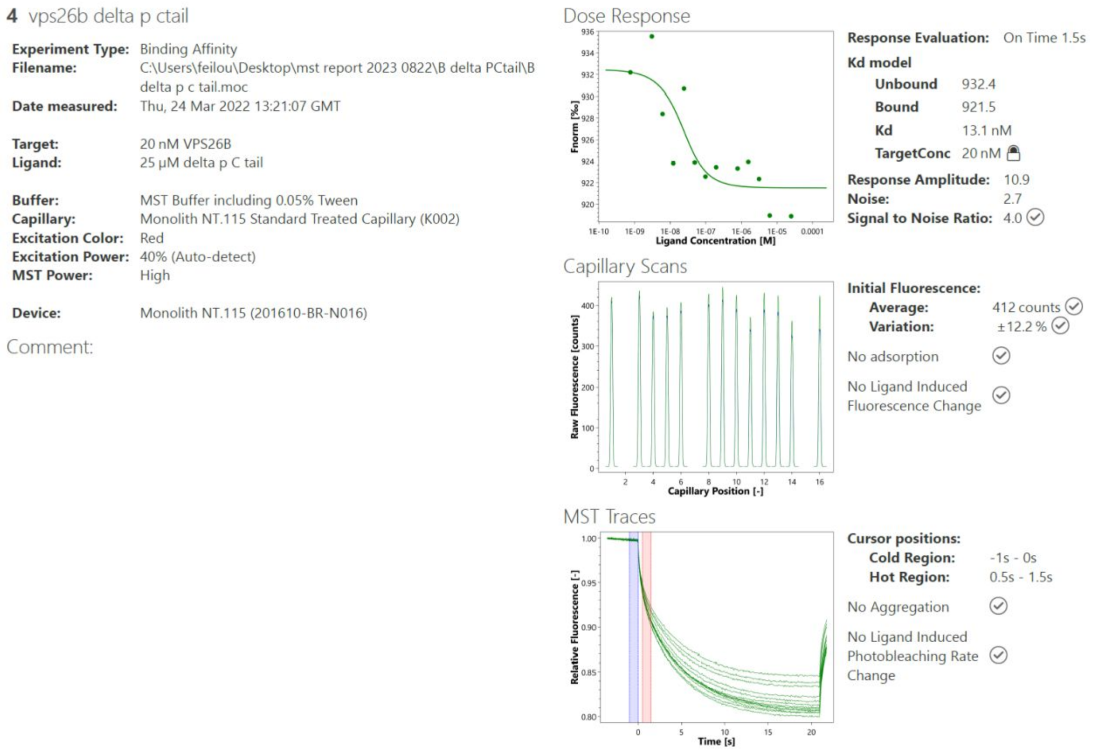

Supplemental Figure S13C “VPS26B → RGS1 delta pC-tail”. Supports Figure 2A

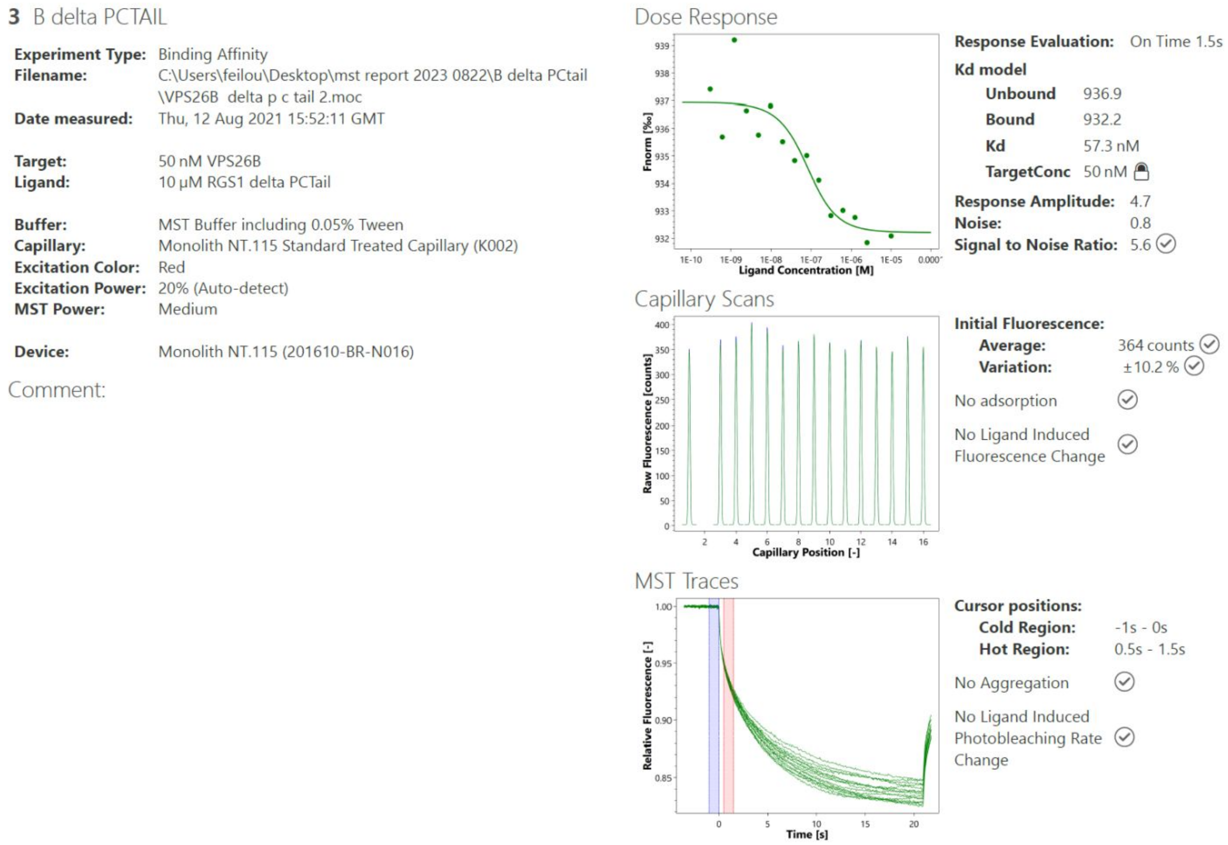

SUPPLEMENTAL INFORMATION

Supplemental Figure S14A “VPS26B → RGS1 R-box + C-tail”. Supports Figure 2A

3 sb j5

Experiment Type: Binding Affinity
Filename: C:\Users\feilou\Desktop\jones\fj\fei\sasbj5bmut\sb j5 ptctl.moc
Date measured: Thu, 24 Mar 2022 13:00:48 GMT

Target: 20 nM VPS26B
Ligand: 17.5 µM RGS1 CTD (j5)

Buffer: MST Buffer including 0.05% Tween
Capillary: Monolith NT.115 Standard Treated Capillary (K002)
Excitation Color: Red
Excitation Power: 20% (Auto-detect)
MST Power: High

Device: Monolith NT.115 (201610-BR-N016)

Comment:

Dose Response

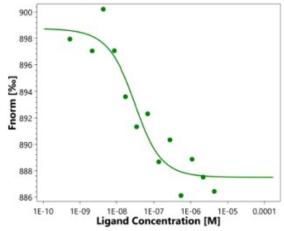

Response Evaluation: On Time 2.5s
Kd model
Unbound 898.7
Bound 887.5
Kd 20.4 nM
TargetConc 20 nM
Response Amplitude: 11.2
Noise: 1.5
Signal to Noise Ratio: 7.7

Capillary Scans

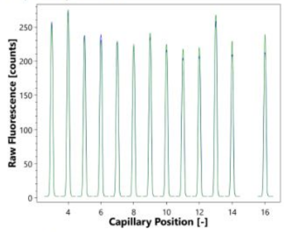

Initial Fluorescence:
Average: 237 counts
Variation: ±15.2 %

No adsorption

No Ligand Induced Fluorescence Change

MST Traces

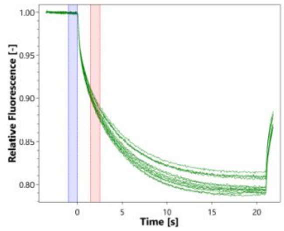

Cursor positions:
Cold Region: -1s - 0s
Hot Region: 1.5s - 2.5s

No Aggregation

No Ligand Induced Photobleaching Rate Change

Supplemental Figure S14B “VPS26B → RGS1 R-box + C-tail”. Supports Figure 2A

5 sb j5

Experiment Type: Binding Affinity
Filename: C:\Users\feilou\Desktop\jones\fj\fei\sasbj5bmut\sb j5 ptctl.moc
Date measured: Thu, 24 Mar 2022 13:41:33 GMT

Target: 20 nM VPS26B
Ligand: 17.5 µM RGS1 CTD (j5)

Buffer: MST Buffer including 0.05% Tween
Capillary: Monolith NT.115 Standard Treated Capillary (K002)
Excitation Color: Red
Excitation Power: 20% (Auto-detect)
MST Power: High

Device: Monolith NT.115 (201610-BR-N016)

Comment:

Dose Response

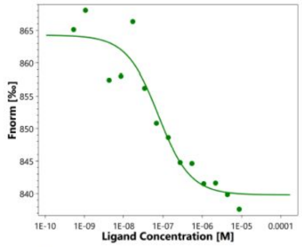

Response Evaluation: On Time 5s
Kd model
Unbound 864.3
Bound 839.8
Kd 66.6 nM
TargetConc 20 nM
Response Amplitude: 24.5
Noise: 3.1
Signal to Noise Ratio: 8.0

Capillary Scans

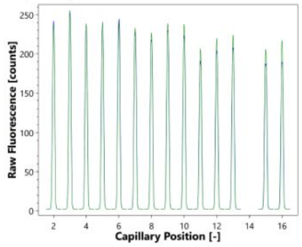

Initial Fluorescence:
Average: 230 counts
Variation: ±10.7 %

No adsorption

No Ligand Induced Fluorescence Change

MST Traces

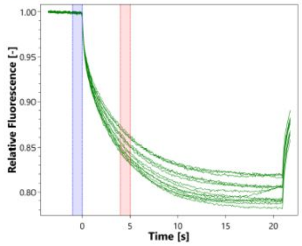

Cursor positions:
Cold Region: -1s - 0s
Hot Region: 4s - 5s

No Aggregation

No Ligand Induced Photobleaching Rate Change

SUPPLEMENTAL INFORMATION

Supplemental Figure S14C "VPS26B → RGS1 R-box + C-tail". Supports Figure 2A

7 sb j5

Experiment Type: Binding Affinity
Filename: C:\Users\feilou\Desktop\jones\fj\fei\sasbj5bmut\sb j5 ptctl.moc
Date measured: Thu, 24 Mar 2022 14:25:22 GMT

Target: 20 nM sb
Ligand: 17.5 µM j5

Buffer: MST Buffer including 0.05% Tween
Capillary: Monolith NT.115 Standard Treated Capillary (K002)
Excitation Color: Red
Excitation Power: 20% (Auto-detect)
MST Power: High

Device: Monolith NT.115 (201610-BR-N016)

Comment:

Dose Response

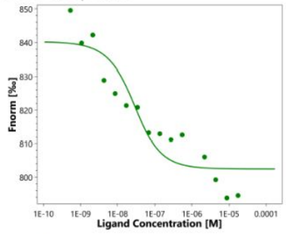

Response Evaluation: On Time 10s
Kd model
Unbound 840.2
Bound 802.4
Kd 18.6 nM
TargetConc 20 nM
Response Amplitude: 37.8
Noise: 6.4
Signal to Noise Ratio: 5.9

Capillary Scans

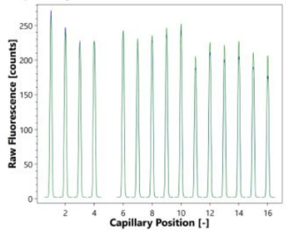

Initial Fluorescence:
Average: 230 counts
Variation: ±14.8 %

No adsorption

No Ligand Induced Fluorescence Change

MST Traces

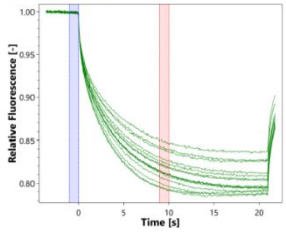

Cursor positions:
Cold Region: -1s - 0s
Hot Region: 9s - 10s

No Aggregation

No Ligand Induced Photobleaching Rate Change

Supplemental Figure S14D "VPS26B → RGS1 R-box + C-tail". Supports Figure 2A

4 Experiment 4

Experiment Type: Binding Affinity
Filename: C:\Users\fei\Desktop\NEW MST DATA\aB J5.moc
Date measured: Wed, 23 Aug 2023 14:05:38 GMT

Target: 20 nM B
Ligand: 20 µM J5

Buffer: MST Buffer including 0.05% Tween
Capillary: Monolith NT.115 Standard Treated Capillary (K002)
Excitation Color: Red
Excitation Power: 100% (Auto-detect)
MST Power: High

Device: Monolith NT.115 (201610-BR-N016)

Comment:

Dose Response

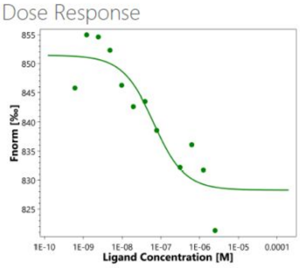

Response Evaluation: On Time 5s
Kd model
Unbound 851.4
Bound 828.3
Kd 55.3 nM
TargetConc 20 nM
Response Amplitude: 23.2
Noise: 4.1
Signal to Noise Ratio: 5.7

Capillary Scans

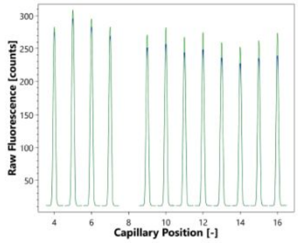

Initial Fluorescence:
Average: 275 counts
Variation: ±12.2 %

No adsorption

Ligand Induced Fluorescence Change
Binding-specific change

MST Traces

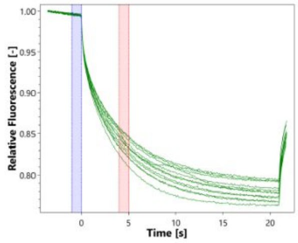

Cursor positions:
Cold Region: -1s - 0s
Hot Region: 4s - 5s

No Aggregation

No Ligand Induced Photobleaching Rate Change

SUPPLEMENTAL INFORMATION

Supplemental Figure S15A "VPS26B → RGS1 pC-tail". Supports Figure 2A

2 bpser2

Experiment Type: Binding Affinity
Filename: C:\Users\feilou\Desktop\mst report 2023 0822\B PCtail\VPS26B p c tail.moc
Date measured: Tue, 17 Aug 2021 12:10:20 GMT

Target: 20 nM VPS26B
Ligand: 5 µM RGS1 p C tail

Buffer: MST Buffer including 0.05% Tween
Capillary: Monolith NT.115 Standard Treated Capillary (K002)
Excitation Color: Red
Excitation Power: 20% (Auto-detect)
MST Power: Medium

Device: Monolith NT.115 (201610-BR-N016)

Comment:

Dose Response

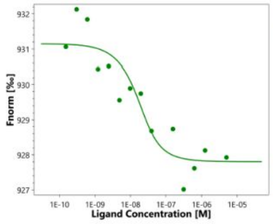

Response Evaluation: On Time 1.5s
Kd model
Unbound 931.2
Bound 927.8
Kd 7.9 nM
TargetConc 20 nM
Response Amplitude: 3.3
Noise: 0.6
Signal to Noise Ratio: 5.5

Capillary Scans

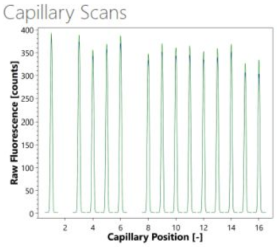

Initial Fluorescence:
Average: 362 counts
Variation: ±9.8 %

No adsorption

No Ligand Induced Fluorescence Change

MST Traces

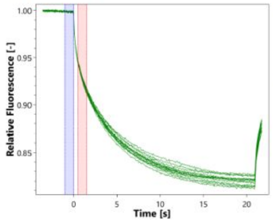

Cursor positions:
Cold Region: -1s - 0s
Hot Region: 0.5s - 1.5s

No Aggregation

No Ligand Induced Photobleaching Rate Change

Supplemental Figure S15B "VPS26B → RGS1 pC-tail". Supports Figure 2A

1 bpser1

Experiment Type: Binding Affinity
Filename: C:\Users\feilou\Desktop\mst report 2023 0822\B PCtail\VPS26B p c tail.moc
Date measured: Tue, 17 Aug 2021 11:46:23 GMT

Target: 20 nM VPS26B
Ligand: 40 µM RGS1 p C tail

Buffer: MST Buffer including 0.05% Tween
Capillary: Monolith NT.115 Standard Treated Capillary (K002)
Excitation Color: Red
Excitation Power: 20% (Auto-detect)
MST Power: Medium

Device: Monolith NT.115 (201610-BR-N016)

Comment:

Dose Response

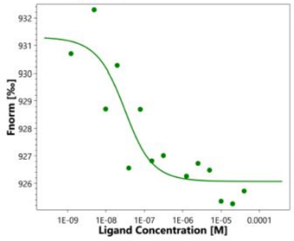

Response Evaluation: On Time 1.5s
Kd model
Unbound 931.3
Bound 926.1
Kd 20.7 nM
TargetConc 20 nM
Response Amplitude: 5.2
Noise: 1.0
Signal to Noise Ratio: 5.3

Capillary Scans

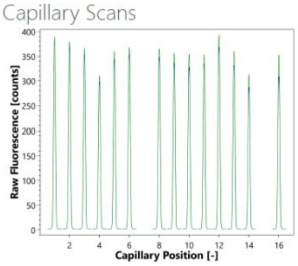

Initial Fluorescence:
Average: 359 counts
Variation: ±13.0 %

No adsorption

No Ligand Induced Fluorescence Change

MST Traces

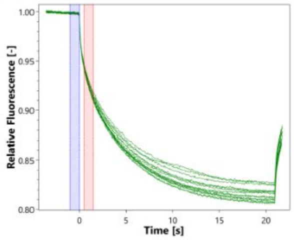

Cursor positions:
Cold Region: -1s - 0s
Hot Region: 0.5s - 1.5s

No Aggregation

No Ligand Induced Photobleaching Rate Change

SUPPLEMENTAL INFORMATION

Supplemental Figure S15C "VPS26B → RGS1 pC-tail". Supports Figure 2A

4 B PCTAIL

Experiment Type: Binding Affinity
Filename: C:\Users\feilou\Desktop\mst report 2023 0822\B PCtail\VPS26B pc tail 2.moc
Date measured: Thu, 12 Aug 2021 16:14:20 GMT

Target: 50 nM vps26b cys
Ligand: 20 µM 3pser

Buffer: MST Buffer including 0.05% Tween
Capillary: Monolith NT.115 Standard Treated Capillary (K002)
Excitation Color: Red
Excitation Power: 20% (Auto-detect)
MST Power: Medium

Device: Monolith NT.115 (201610-BR-N016)

Comment:

Dose Response

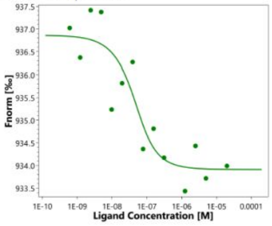

Response Evaluation: On Time 1.5s
Kd model
Unbound 936.9
Bound 933.9
Kd 22.1 nM
TargetConc 50 nM
Response Amplitude: 3.0
Noise: 0.6
Signal to Noise Ratio: 5.1

Capillary Scans

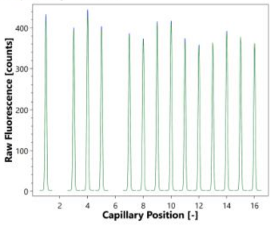

Initial Fluorescence:
Average: 387 counts
Variation: ±11.1 %
No adsorption
No Ligand Induced Fluorescence Change

MST Traces

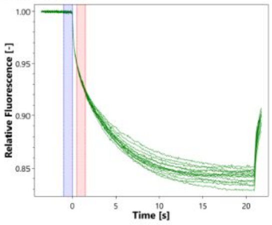

Cursor positions:
Cold Region: -1s - 0s
Hot Region: 0.5s - 1.5s
No Aggregation
No Ligand Induced Photobleaching Rate Change

Supplemental Figure S16 "VPS26A → C domain no tail". Supports Figure 2A

6 aj7

Experiment Type: Binding Affinity
Filename: C:\Users\fei\Desktop\desktop\MST DATA\old data vps pser \ab.moc
Date measured: Fri, 03 Sep 2021 15:29:07 GMT

Target: 20 nM VPS26A
Ligand: 5 µM RGS1 C domain no tail

Buffer: MST Buffer including 0.05% Tween
Capillary: Monolith NT.115 Standard Treated Capillary (K002)
Excitation Color: Red
Excitation Power: 20% (Auto-detect)
MST Power: Medium

Device: Monolith NT.115 (201610-BR-N016)

Comment:

Dose Response

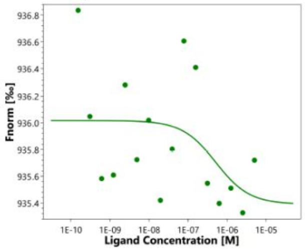

Response Evaluation: On Time 1.5s
Kd model
Unbound 936
Bound 935.4
Kd 502 nM
TargetConc 20 nM
Response Amplitude: 0.6
Noise: 0.4
Signal to Noise Ratio: 1.5

Capillary Scans

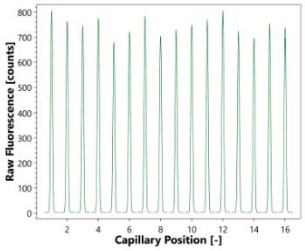

Initial Fluorescence:
Average: 746 counts
Variation: ±8.6 %
No adsorption
No Ligand Induced Fluorescence Change

MST Traces

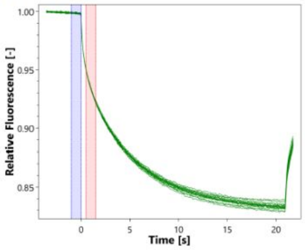

Cursor positions:
Cold Region: -1s - 0s
Hot Region: 0.5s - 1.5s
No Aggregation
No Ligand Induced Photobleaching Rate Change

SUPPLEMENTAL INFORMATION

Supplemental Figure S17 “VPS26B → C domain no tail”. Supports Figure 2A

2 bj7

Experiment Type: Binding Affinity
Filename: C:\Users\fei\Desktop\desktop\abj7.moc
Date measured: Thu, 09 Sep 2021 13:42:31 GMT

Target: 20 nM VPS26B
Ligand: 5 µM RGS1 C doamin no tail

Buffer: MST Buffer including 0.05% Tween
Capillary: Monolith NT.115 Standard Treated Capillary (K002)
Excitation Color: Red
Excitation Power: 40% (Auto-detect)
MST Power: Medium

Device: Monolith NT.115 (201610-BR-N016)

Comment:

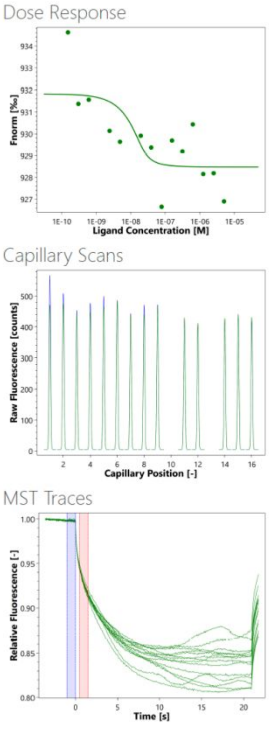

Response Evaluation: On Time 1.5s
Kd model
Unbound 931.8
Bound 928.5
Kd 2.37 nM
TargetConc 20 nM
Response Amplitude: 3.3
Noise: 1.4
Signal to Noise Ratio: 2.4

Initial Fluorescence:
Average: 448 counts
Variation: ±8.5 %
No adsorption
No Ligand Induced Fluorescence Change

Cursor positions:
Cold Region: -1s - 0s
Hot Region: 0.5s - 1.5s
No Aggregation
No Ligand Induced Photobleaching Rate Change

Supplemental Figure S18 “VPS26A → VPS26A”. Supports Figure 2A

4 aa

Experiment Type: Binding Affinity
Filename: C:\Users\fei\Desktop\desktop\MST DATA\old data vps pser \ab.moc
Date measured: Fri, 03 Sep 2021 14:36:54 GMT

Target: 20 nM VPS26A
Ligand: 15 µM VPS26A

Buffer: MST Buffer including 0.05% Tween
Capillary: Monolith NT.115 Standard Treated Capillary (K002)
Excitation Color: Red
Excitation Power: 20% (Auto-detect)
MST Power: Medium

Device: Monolith NT.115 (201610-BR-N016)

Comment:

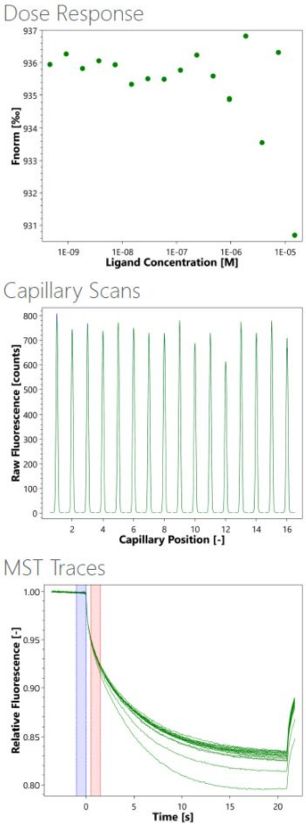

Response Evaluation: On Time 1.5s
Kd model
Unbound N/A
Bound N/A
Kd N/A
TargetConc 20 nM
Response Amplitude: N/A
Noise: N/A
Signal to Noise Ratio: N/A

Initial Fluorescence:
Average: 737 counts
Variation: ±16.6 %
No adsorption
No Ligand Induced Fluorescence Change

Cursor positions:
Cold Region: -1s - 0s
Hot Region: 0.5s - 1.5s
No Aggregation
No Ligand Induced Photobleaching Rate Change

Supplemental Figure S19 "VPS26B → VPS26B". Supports Figure 2A

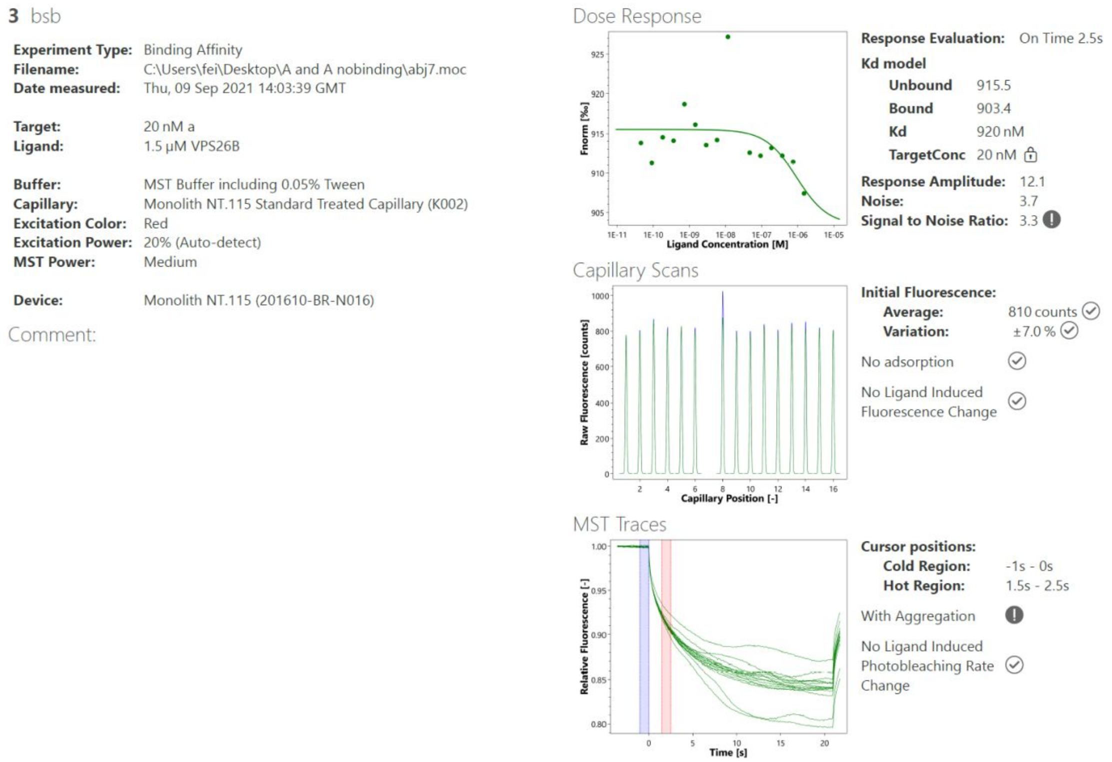

Supplemental Figure S20A "VPS26AB → RGS1 pC-tail". Supports Figure 2A

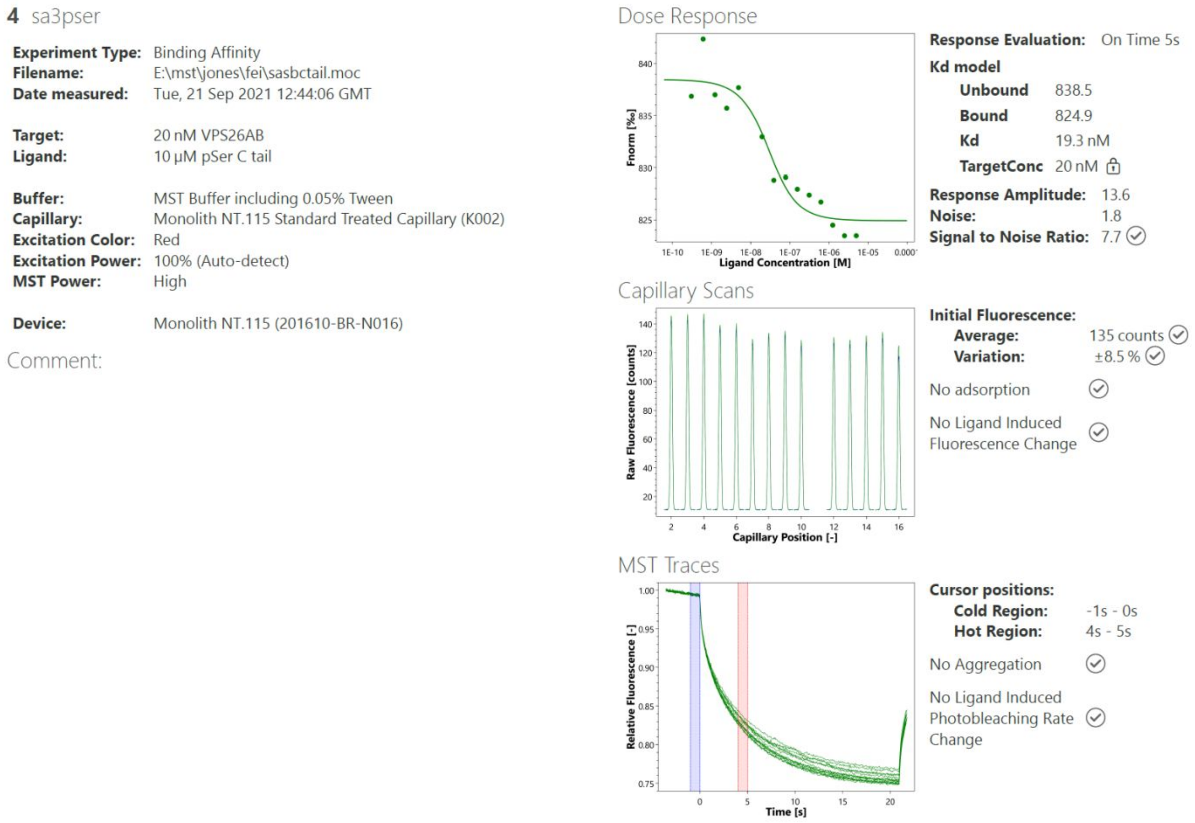

SUPPLEMENTAL INFORMATION

Supplemental Figure S20B “VPS26AB → RGS1 pC-tail”. Supports Figure 2A

7 sactail

**Experiment Type:**	Binding Affinity
**Filename:**	E:\mst\jones\fei\sasbctail.moc
**Date measured:**	Tue, 21 Sep 2021 14:10:09 GMT
**Target:**	20 nM VPS26AB heterodimer
**Ligand:**	50 µM pSer C tail
**Buffer:**	MST Buffer including 0.05% Tween
**Capillary:**	Monolith NT.115 Standard Treated Capillary (K002)
**Excitation Color:**	Red
**Excitation Power:**	100% (Auto-detect)
**MST Power:**	High
**Device:**	Monolith NT.115 (201610-BR-N016)

Comment:

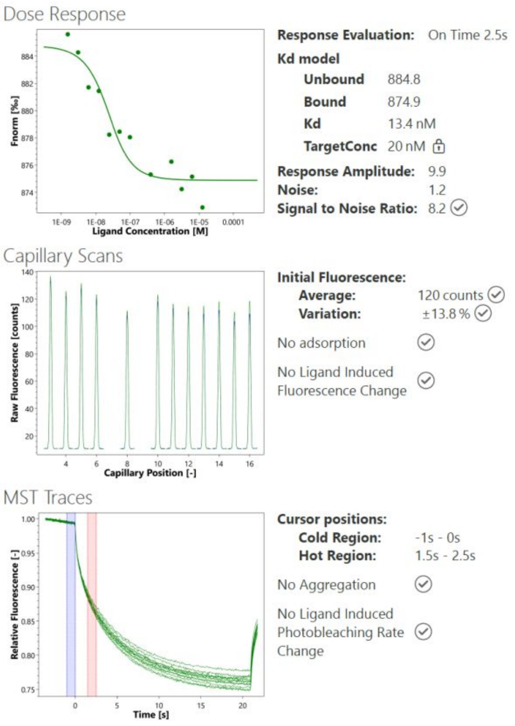

Supplemental Figure S20C “VPS26AB → RGS1 pC-tail”. Supports Figure 2A

6 sasbser2

**Experiment Type:**	Binding Affinity
**Filename:**	E:\mst\jones\fei\sasbctail.moc
**Date measured:**	Tue, 21 Sep 2021 13:49:40 GMT
**Target:**	20 nM VPS26AB heterodimer
**Ligand:**	10 µM pSer C tail
**Buffer:**	MST Buffer including 0.05% Tween
**Capillary:**	Monolith NT.115 Standard Treated Capillary (K002)
**Excitation Color:**	Red
**Excitation Power:**	100% (Auto-detect)
**MST Power:**	High
**Device:**	Monolith NT.115 (201610-BR-N016)

Comment:

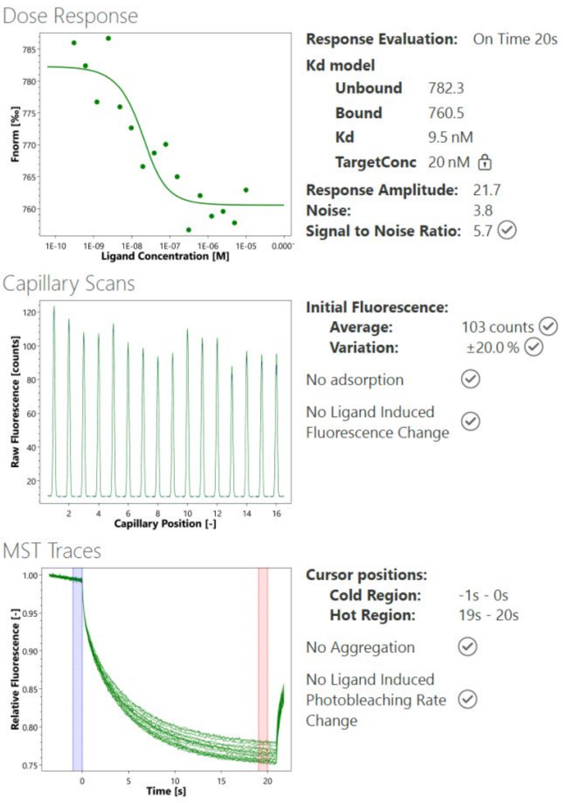

SUPPLEMENTAL INFORMATION

Supplemental Figure S20D "VPS26AB → RGS1 pC-tail". Supports Figure 2A

sasbser2

Experiment Type: Binding Affinity
Filename: E:\mst\jones\fei\sasbctail.moc
Date measured: Tuesday Sep 21 15:20:40 GMT

Target: 20 nM VPS26AB heterodimer
Ligand: 10 µM pSer C tail

Buffer: MST Buffer including 0.05% Tween
Capillary: Monolith NT.115 Standard Treated Capillary (K002)
Excitation Color: Red
Excitation Power: 100% (Auto-detect)
MST Power: High

Device: Monolith NT.115 (201610-BR-N016)

Comment:

Dose Response

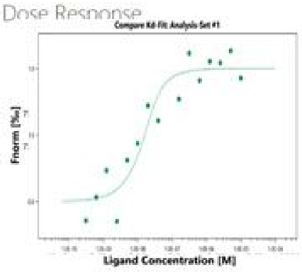

Response Evaluation: On Time 20s
Kd model
Unbound 783.47
Bound 761.09
Kd 5.749 +/- 3.47
TargetConc 20 nM
Response Amplitude: 22.379
Noise:
Signal to Noise Ratio: 6.96

Capillary Scans

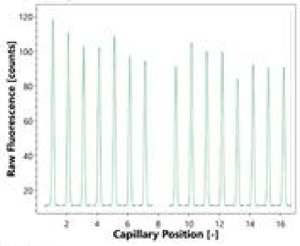

Initial Fluorescence:
Average: 103 counts
Variation: ±20.0 %
No adsorption
No Ligand Induced Fluorescence Change

MST Traces

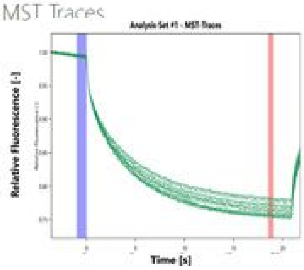

Cursor positions:
Cold Region: -1s - 0s
Hot Region: 18.5s - 19.07s
No Aggregation
No Ligand Induced Photobleaching Rate Change
